# Hypoimmune induced pluripotent stem cells survive long term in fully immunocompetent, allogeneic rhesus macaques

**DOI:** 10.1038/s41587-023-01784-x

**Published:** 2023-05-08

**Authors:** Xiaomeng Hu, Kathy White, Ari G. Olroyd, Rowena DeJesus, Antonia A. Dominguez, William E. Dowdle, Annabelle M. Friera, Chi Young, Frank Wells, Elaine Y. Chu, Cade Ellis Ito, Harini Krishnapura, Surbhi Jain, Ramya Ankala, Trevor J. McGill, August Lin, Kyla Egenberger, Allison Gagnon, J. Michael Rukstalis, Nathaniel J. Hogrebe, Corie Gattis, Ron Basco, Jeffrey R. Millman, Paul Kievit, Mark M. Davis, Lewis L. Lanier, Andrew J. Connolly, Tobias Deuse, Sonja Schrepfer

**Affiliations:** 1 Sana Biotechnology, Inc., South San Francisco, CA USA; 2 Division of Endocrinology, Metabolism and Lipid Research, Washington University School of Medicine, St. Louis, MO USA; 3 Division of Cardiometabolic Health, Oregon National Primate Research Center, Beaverton, OR USA; 4 Howard Hughes Medical Institute, Institute for Immunity, Transplantation and Infection, and Department of Microbiology and Immunology, Stanford University School of Medicine, Stanford, CA USA; 5 Department of Microbiology and Immunology and the Parker Institute for Cancer Immunotherapy, University of California, San Francisco, San Francisco, CA USA; 6 Department of Pathology, University of California, San Francisco, San Francisco, CA USA; 7 Transplant and Stem Cell Immunobiology (TSI) Lab, Department of Surgery, Division of Cardiothoracic Surgery, University of California, San Francisco, San Francisco, CA USA

**Keywords:** Stem-cell biotechnology, Stem-cell research

## Abstract

Genetic engineering of allogeneic cell therapeutics that fully prevents rejection by a recipient’s immune system would abolish the requirement for immunosuppressive drugs or encapsulation and support large-scale manufacturing of off-the-shelf cell products. Previously, we generated mouse and human hypoimmune pluripotent (HIP) stem cells by depleting HLA class I and II molecules and overexpressing CD47 (*B2M*^*−/−*^*CIITA*^*−/−*^CD47^*+*^). To determine whether this strategy is successful in non-human primates, we engineered rhesus macaque HIP cells and transplanted them intramuscularly into four allogeneic rhesus macaques. The HIP cells survived unrestricted for 16 weeks in fully immunocompetent allogeneic recipients and differentiated into several lineages, whereas allogeneic wild-type cells were vigorously rejected. We also differentiated human HIP cells into endocrinologically active pancreatic islet cells and showed that they survived in immunocompetent, allogeneic diabetic humanized mice for 4 weeks and ameliorated diabetes. HIP-edited primary rhesus macaque islets survived for 40 weeks in an allogeneic rhesus macaque recipient without immunosuppression, whereas unedited islets were quickly rejected.

## Abstract (web summary)

A genetic method to block immune rejection of allogeneic cells is validated in monkeys.

## Main

Regenerative cell therapeutics derived from induced pluripotent stem cells (iPSCs) have enormous potential for treating diseases characterized by the loss of specialized cell populations^1^. Insulin-producing β cells derived from iPSCs^2,3^ and manufactured at scale^4^ suggest a pathway to clinical islet cell replacement therapy in patients with diabetes mellitus. However, a remaining challenge is achieving efficient survival, engraftment and long-term functionality of transplanted cells. Although individualized, patient-specific autologous approaches^5^, encapsulation of allogeneic cells^6^ or heavy immunosuppression of cell recipients^7^ have been proposed, such approaches are naturally limited by scalability, batch variability, quality control, regimen side effects, toxicity of the encapsulation or functionality. Therefore, the generation of allogeneic, immune-evasive islet cells might allow greater patient access to such therapies.

The expression of highly polymorphic human leukocyte antigens (HLAs) defines the tissue histocompatibility type, and, whereas HLA class I molecules are expressed on all nucleated cells, class II complexes are restricted to antigen-presenting cells (APCs) and some non-professional APCs^8^. The depletion of class I and II HLAs via gene editing is aimed at broadly avoiding lymphocyte recognition^9^ and can be achieved by inactivating *B2M* and one of *CIITA*, *RFX5*, *RFXANK* or *RFXAP* genes. *B2M* inactivation, however, triggers a ‘missing self’ killing response^10^ by natural killer (NK) cells and macrophages, both of which recognize HLA class I deficiency^11^. The opposing functionality of HLA class I has sparked ideas for inhibiting this ‘missing self’ response that can be divided into HLA engineering and immune checkpoint inhibition. HLA-E and HLA-G bind the inhibitory NK cell receptors CD94/NKG2A^12^ and LILRB1 (ILT2)^13^, and forced expression of monomorphic HLA-E^14^ and HLA-G^15^ on graft cells has been proposed as HLA engineering strategies. However, because HLA-E and HLA-G require the association with B2M, which is missing in *B2M*^*−/−*^ cells, this requires a fusion construct to link the HLA heavy chain to a transgenic B2M^14^. Overexpression of the immune checkpoint inhibitors PD-L1 and CD47 (ref. ^16^), which bind PD-1 (ref. ^17^) and signal regulatory protein (SIRP) α^11^, respectively, has alternatively been proposed for the HLA-independent inhibition of innate immune cells.

We compared these engineering approaches and show that our *B2M*^−/−^*CIITA*^−/−^CD47^+^ HIP immune editing effectively prevents allogeneic adaptive and innate immune activation. We further show that HIP editing can be applied to iPSCs and specialized primary cells, and we confirmed their immune-evasive nature in non-human primates. Rhesus macaque HIP iPSCs and HIP pancreatic islet cells achieved long-term survival in fully allogeneic immunocompetent recipients. HIP editing may allow large-scale manufacturing of universal cell-based therapies.

## Results

### CD47 overexpression is a reliable immune checkpoint inhibitor for NK cells

We performed a head-to-head comparison of HLA engineering and immune checkpoint inhibition strategies in a simplified experimental setup with K562, a naturally HLA class I- and II-deficient cell line that expresses B2M. Transgenes for HLA-E, HLA-G, PD-L1 or CD47 were selectively overexpressed (Supplementary Fig. 1a–e). NK cells were stimulated with IL-2, a key cytokine associated with allograft rejection^18^. Human T cells, K562 and the transgenic K562 derivatives were injected intramuscularly into humanized NSG-SGM3 mice, and, after 6 d, spleen and serum were recovered (Supplementary Fig. 1f). ELISpot assays with recipient splenocytes and allogeneic T cells generated high interferon (IFN)-γ spot frequencies and, thus, confirmed the robustness of the humanized immune system in humanized NSG-SGM3 recipients (Supplementary Fig. 1g). K562^HLA-E^ and K562^HLA-G^ triggered a T cell response with approximately half the spot frequencies of those with allogeneic T cells. Unmodified K562 and K562^PD-L1^ and K562^CD47^ did not induce any measurable IFN-γ response. The serum was screened for donor cell-specific antibodies (DSAs), and the injection of allogeneic T cells resulted in a surge of IgM antibodies binding these allogeneic T cells (Supplementary Fig. 1h). K562^HLA-E^ and K562^HLA-G^ also induced IgM DSAs, although at lower levels. K562 and K562^PD-L1^ and K562^CD47^ did not induce any IgM DSAs. Together, these data show that transgenic expression of monomorphic HLA-E or HLA-G does not prevent T cell activation and DSA generation.

Next, we assessed the ability of the four editing strategies to comprehensively inhibit killing of HLA class I-deficient cells by IL-2-stimulated human peripheral blood NK cells. Most isolated NK cells were CD56^dim^, with fewer CD56^bright^ cells and minimal CD3^+^ contamination (Supplementary Fig. 2a,b). K562 or transgenic K562 target cells were grown on special electrode plates for real-time impedance assays. A drop in impedance reflects an increase in electric current between biosensors from a breakdown of the target cell layer. Only 31.4% of NK cells expressed the HLA-E receptor subunit CD94 (Supplementary Fig. 2c). K562^HLA-E^ were resistant against sorted NK cells expressing CD94 but were swiftly killed by CD94^−^ NK cells and by a mixed, unsorted NK cell population (Supplementary Fig. 2d). Accordingly, CD94^+^ NK cells did not release granzyme B or perforin when incubated with K562^HLA-E^, whereas CD94^−^ NK and unsorted NK cells showed strong granule protein release (Supplementary Fig. 2e). We found that 62.0% of human NK cells express the HLA-G receptor ILT2, and only ILT2^+^ NK cells were unable to kill K562^HLA-G^ (Supplementary Fig. 2f–h). The leader sequence of HLA-G contains a non-americ peptide capable of stabilizing HLA-E, and co-expression of functional endogenous HLA-E in HLA-G-transfected 721.221 has been reported^13^. In K562^HLA-G^, we did not observe co-expression of HLA-E, and CD94^+^ILT2^−^ NK cells killed the K562^HLA-G^ rapidly (Supplementary Fig. 2i–k). The expression of ILT2 was required to suppress NK cell killing of K562^HLA-G^, and CD94 expression was not relevant. Only 1.3% of NK cells expressed PD-1, and only PD-1^+^ NK cells spared K562^PD-L1^ (Supplementary Fig. 2l–n). Notably, almost all IL-2-activated NK cells showed SIRPα expression, and K562^CD47^ were comprehensively protected from all NK cell killing (Supplementary Fig. 2o–q). In confirmatory in vivo NK cell killing experiments, human T cells and one of the K562 lines were simultaneously injected into the peritoneum of NSG mice at a 1:1 ratio. Sorted or unsorted human NK cells were adoptively transferred. Although HLA-expressing T cells did not undergo NK cell clearance in this model, K562 were largely eliminated over 48 h (Supplementary Fig. 2r). K562^HLA-E^, K562^HLA-G^ and K562^PD-L1^ were spared only by NK cells expressing the corresponding inhibitory receptor, whereas K562^CD47^ was completely protected from in vivo NK cell killing (Supplementary Fig. 2s–v). In summary, only K562 with CD47 overexpression prevented both T cell activation and NK cell killing in vitro and in vivo.

### Human HIP cells avoid immune activation and systemic rejection in rhesus macaques

To assess the hypoimmune editing platform in clinically relevant scenarios, we studied the immune response against HIP in non-human primates. We designed a two-pronged study testing xenogeneic human iPSCs, because they may be used as source for future human cell therapeutics and rhesus macaque iPSCs as allogeneic analogs. First, human wild-type (wt^xeno^) iPSCs underwent CRISPR–Cas9 inactivation of *B2M* and *CIITA* and were transduced with lentiviral particles carrying a transgene of macaque CD47 to generate HIP^xeno^ (Supplementary Fig. 3a–d). Previous studies showed the species specificity of the CD47–SIRPα axis^11^. Both iPSCs were transduced to express firefly luciferase (FLuc) for bioluminescence imaging (BLI) to track viable cell numbers. Human wt^xeno^ and HIP^xeno^ iPSCs showed good viability at the time of cell injection (Supplementary Fig. 3e). When injected into the thigh muscle of NSG mice, both wt^xeno^ and HIP^xeno^ iPSCs showed similar proliferation (Supplementary Fig. 3f–g) and formed palpable tumors, histologically identified as teratomas (Supplementary Fig. 4).

Four rhesus macaques received four subcutaneous injections on their backs, each with 10 million human wt^xeno^ or HIP^xeno^ iPSCs. They received a second injection of the same iPSCs on day 75 and, in a cross-over design, a third injection of the other iPSCs after another 75 d. The first reinjection of the same iPSC was meant to study the immune response after re-dosing of the same cell product, and the cross-over reinjection was meant to study whether sensitization with one iPSC population has implications on the response against the other iPSCs. All rhesus macaques had blood drawn in regular intervals for comprehensive immune assays. Peripheral blood mononuclear cells (PBMCs) were isolated for ELISpot assays, and killing assays were performed with PBMCs, T cells, NK cells and PBMC-derived macrophages (Supplementary Fig. 5). Graphs show the percent target cell killing after 90 h (circles) as well as the speed of killing (triangles, (time to cell index of 0.5)^−1^). There was no pre-existing cellular reactivity against wt^xeno^ or HIP^xeno^, and we saw no target cell killing by immune cells drawn before the cell transplantation (day 0). In the group receiving wt^xeno^ first, there was strong T cell activation with ELISpot frequencies around 600 on day 7 (Supplementary Fig. 5a) and rapid in vitro wt^xeno^ killing by PBMCs and T cells (Supplementary Fig. 5b,c). ELISpot frequencies came down markedly over 75 d, and the speed of killing by PBMCs and T cells came down accordingly. Re-injection of wt^xeno^ on day 75 induced a second response with similar magnitude and kinetics. HIP^xeno^, injected after another 75 d, did not experience any measurable cellular immune response despite the two previous sensitization events with wt^xeno^. As expected, we did not observe any killing of HLA-expressing wt^xeno^ by NK cells or macrophages (Supplementary Fig. 5d,e). More remarkably, we also never observed any killing of HLA-deficient HIP^xeno^ by NK cells and macrophages, thus confirming the protective efficacy of rhesus CD47 in monkeys. Rhesus macaques that received HLA class I- and II-deficient human iPSCs (double knockout (DKO)) without rhesus CD47 overexpression showed swift NK cell and macrophage killing (Supplementary Fig. 6). In the group receiving HIP^xeno^ first, there was no cellular immune activation or killing by immune cells after the first and second injection (Supplementary Fig. 5f–j). The subsequently injected wt^xeno^ induced strong cellular immune responses similar to those observed in the group receiving wt^xeno^ first and showed that HIP^xeno^ does not exert any immunosuppressive effects extending to non-HIP cells.

Rhesus macaque serum drawn at the same timepoints was screened for antibodies enabling antibody-dependent cellular cytotoxicity (ADCC) with rhesus macaque NK cells and macrophages or complement-dependent cytotoxicity (CDC; Supplementary Fig. 7). In the group receiving wt^xeno^ first, we observed an early peak of total IgM antibodies after 7 d and a delayed peak of total IgG by day 13 (Supplementary Fig. 7a,b). DSA levels for IgM and IgG followed the same kinetics, hinting at the specificity of the antibodies produced (Supplementary Fig. 7c,d). ADCC and CDC assays were remarkable for the existence of pre-formed anti-wt^xeno^ antibodies (Supplementary Fig. 7e–g). However, although there was killing at all timepoints, the increase in DSA levels after the first and second wt^xeno^ injection increased the speed of wt^xeno^ killing. Despite the existence of pre-formed anti-wt^xeno^ antibodies and two sensitizing wt^xeno^ transplant events, the subsequently transplanted HIP^xeno^ did not induce DSAs and did not undergo ADCC or CDC killing. In the group receiving HIP^xeno^ first, there was no increase in antibody levels and no HIP^xeno^ killing in all assays. The rhesus macaques, however, mounted a vigorous antibody and killing response against the subsequently transplanted wt^xeno^ (Supplementary Fig. 7h–n). Although easily accessible and having adequate tissue volume capacity^19^, cell transplantation into an unmodified subcutaneous site in large animals and humans has repeatedly been shown not to support cell survival as it is an inhospitable microenvironment owing to poor oxygen tension and inadequate vascularization^20,21^. For those reasons, we switched to an intramuscular injection site for the subsequent allogeneic cell transplant experiments.

### Allogeneic HIP cells avoid immune activation and rejection in rhesus macaques

To evaluate the potency of the HIP approach without the constraints of a xenogeneic transplant setting, rhesus macaque HIP cells were generated. We performed CRISPR–Cas9 inactivation of the *B2M* and *CIITA* genes in rhesus macaque wt^allo^ iPSCs, followed by lentiviral overexpression of rhesus CD47. Two rhesus macaque HIP^allo^ iPSC clones were selected and confirmed to have the hypoimmune phenotype of major histocompatibility complex (MHC) deficiency and CD47 overexpression (Supplementary Fig. 8a–f). All iPSCs were transduced to express FLuc for BLI imaging. Cells were harvested and showed good viability (Supplementary Fig. 8g). Ten million cells were injected into NSG mice, and wt^allo^ and HIP^allo^ clones 1B4 and 2B5 showed similar proliferation characteristics. However, when compared to their human counterparts studied before, 10 times more rhesus macaque iPSCs had to be injected to form teratomas, and their proliferation was markedly slower (Supplementary Figs. 8h–j and 9).

**Fig. 1:**
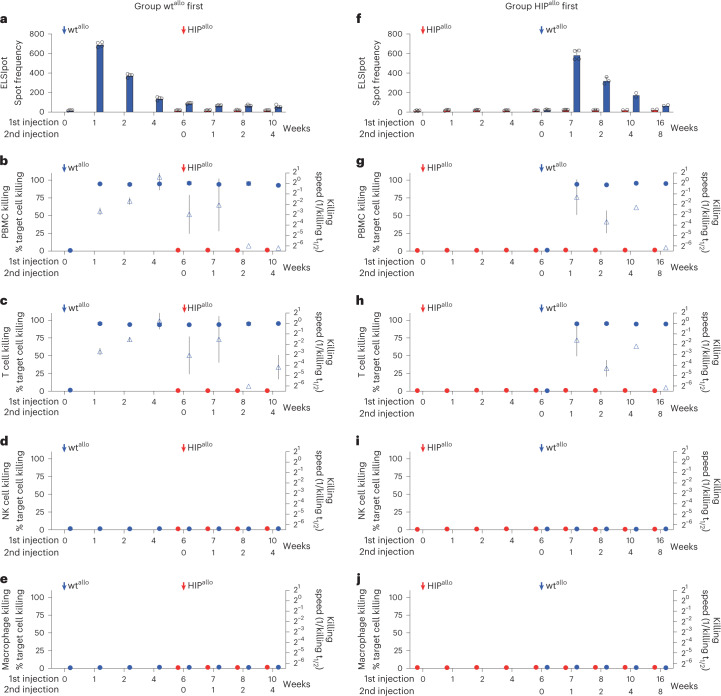
Cellular immune responses against allogeneic rhesus macaque wt and HIP grafts. **a**–**e**, Immune assays in the group receiving wt^allo^ first. ELISpot assays with recipient monkey PBMCs drawn at scheduled timepoints (**a**, mean ± s.d., four monkeys). Killing assays with recipient monkey PBMCs (**b**), T cells (**c**), NK cells (**d**) and macrophages (**e**). Percent target cell killing is shown on the left *y* axis (mean ± s.d.), and killing speed is shown on the right *y* axis (killing t_1/2_^−1^, mean ± s.e.m., four monkeys). **f**–**j**, Immune assays in the group receiving HIP^allo^ first. ELISpot assays with recipient monkey PBMCs (**f**, mean ± s.d., four monkeys at weeks 0–7, three monkeys at week 8 and two monkeys at weeks 10–16). Killing assays with recipient monkey PBMCs (**g**), T cells (**h**), NK cells (**i**) and macrophages (**j**). Percent target cell killing is shown on the left *y* axis (mean ± s.d.), and killing speed is shown on the right *y* axis (killing t_1/2_^−1^, mean ± s.e.m., four monkeys at weeks 0–7, three monkeys at week 8 and two monkeys at weeks 10–16). All assays run against wt^allo^ and HIP^allo^ are shown in blue and red, respectively, at 6 weeks, and, later, all assays were run against both cell types.

To better support cell survival and long-term growth, the wt^allo^ and HIP^allo^ rhesus macaque iPSCs were injected intramuscularly into the quadriceps muscle of allogeneic rhesus macaques, which is very well vascularized. Each group consisted of four animals that received wt^allo^ or HIP^allo^ first and the other iPSCs after 6 weeks into the contralateral leg. Animals had regular blood draws for immune assays and BLI imaging to quantitatively assess graft survival. Grafts were recovered at different timepoints for histology. Cellular immune assays did not show any pre-existing responsiveness (Fig. 1). The group receiving wt^allo^ first mounted a strong T cell response with high ELISpot frequencies after 1 week and rapid killing by PBMCs and T cells (Fig. 1a–c). When HIP^allo^ were injected 6 weeks later, the monkeys continued to show a strong cellular response against wt^allo^ but showed no reactivity against HIP^allo^ in all assays. HIP^allo^ elicited no killing by NK cells and macrophages (Fig. 1d,e), which confirmed the efficacy of the hypoimmune concept in rhesus macaque cells. In the group receiving HIP^allo^ first, there was no measurable cellular immune response against HIP^allo^ and continued to be no response even after wt^allo^ were injected and the monkeys mounted a strong response against wt^allo^ (Fig. 1f–j).

**Fig. 2:**
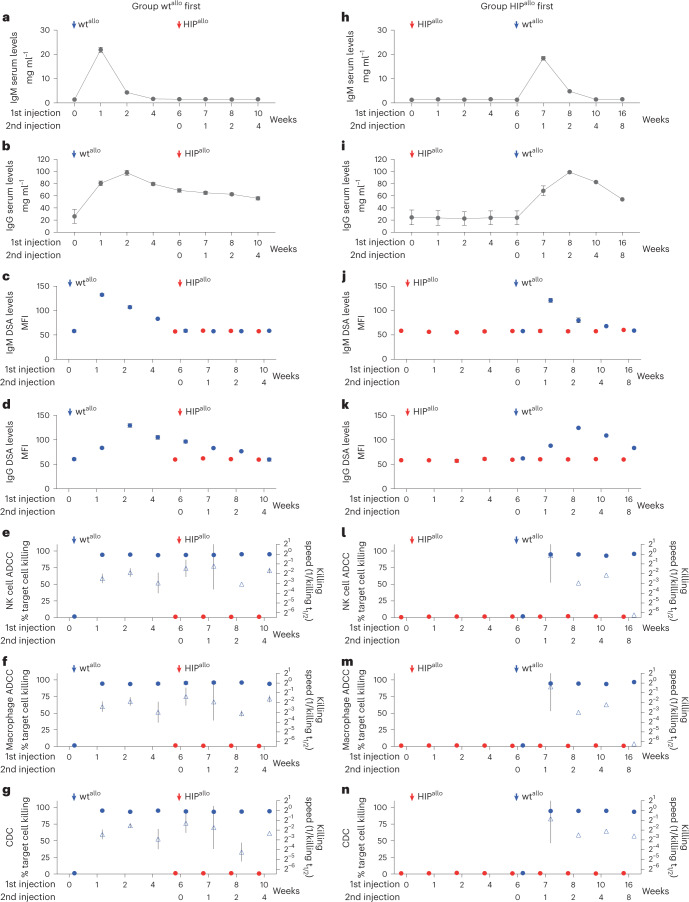
Antibody-mediated responses against allogeneic rhesus macaque wt and HIP grafts. **a**–**g**, Antibody immune assays in the group receiving wt^allo^ first. Total serum IgM (**a**) and IgG (**b**) levels and DSA IgM (**c**) and IgG (**d**) levels are shown (mean ± s.d., four monkeys). ADCC assays with de-complemented recipient monkey serum and NK cells (**e**) or macrophages (**f**) and CDC assays with complete recipient monkey serum (**g**) are shown. Percent target cell killing is shown on the left *y* axis (mean ± s.d.), and killing speed is shown on the right *y* axis (killing t_1/2_^−1^, mean ± s.e.m., four monkeys). **h**–**n**, Antibody immune assays in the group receiving HIP^allo^ first. Total serum IgM (**h**) and IgG (**i**) levels and DSA IgM (**j**) and IgG (**k**) levels are shown (mean ± s.d., four monkeys at weeks 0–7, three monkeys at week 8 and two monkeys at weeks 10–16). ADCC assays with de-complemented recipient monkey serum and NK cells (**l**) or macrophages (**m**) and CDC assays with complete recipient monkey serum (**n**) are shown. Percent target cell killing is shown on the left *y* axis (mean ± s.d.), and killing speed is shown on the right *y* axis (killing t_1/2_^–1^, mean ± s.e.m., four monkeys at weeks 0–7, three monkeys week at 8 and two monkeys at weeks 10–16). All assays run against wt^allo^ and HIP^allo^ are shown in blue and red, respectively, at 6 weeks, and, later, all assays were run against both cell types.

Antibody assays showed a sharp peak of IgM antibodies (Fig. 2a) in the group receiving wt^allo^ first, followed by a later surge of IgG antibodies with a slow decline (Fig. 2b). The total antibody levels correlated well with IgM and IgG DSA levels (Fig. 2c–d). The recipients had no pre-formed antibodies against the allogeneic wt^allo^ cells, and we saw no pre-transplant ADCC or CDC. The recipient animals, however, exerted rapid ADCC and CDC against wt^allo^ from the first week until the end of the study (Fig. 2e–g). However, there was no antibody response, ADCC or CDC against HIP^allo^ after they were injected at 6 weeks. In the group receiving HIP^allo^ first, there was no increase in antibody levels, no DSAs and no signs of ADCC or CDC against HIP^allo^ (Fig. 2h–n). After wt^allo^ was injected at 6 weeks, we saw a strong antibody and killing response against wt^allo^, which was very similar to that in the group receiving wt^allo^ first. Despite the strong antibody response against wt^allo^, there was still no measurable immune activity against HIP^allo^.

### Allogeneic HIP cells achieve long-term survival in rhesus macaques

**Fig. 3:**
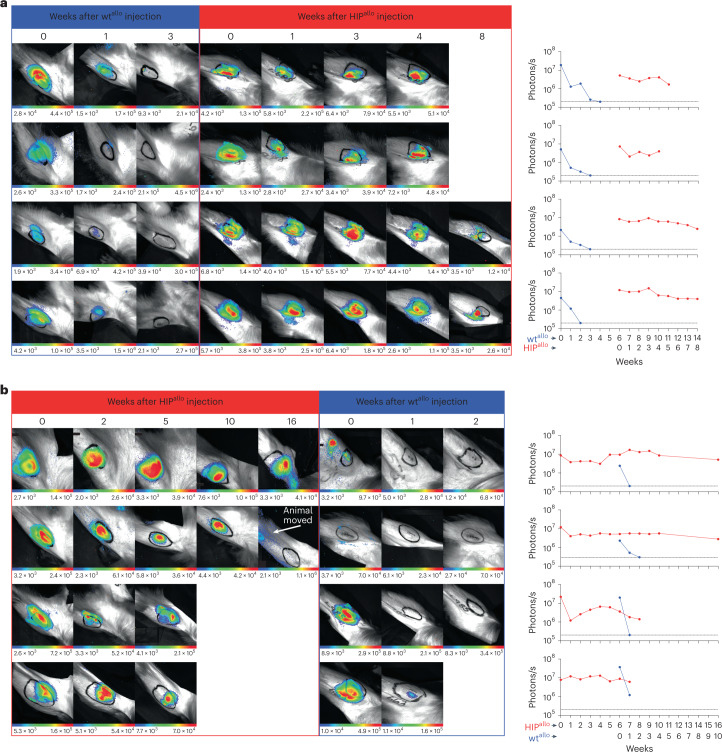
Graft survival assessed by quantitative BLI. **a**,**b**, BLI images and BLI signals over time are shown for all four rhesus macaques receiving wt^allo^ first (**a**) and all four monkeys receiving HIP^allo^ first (**b**).

Cell survival was quantitatively followed by BLI imaging. In the group receiving wt^allo^ first, the BLI signals diminished quickly, and graft viability was completely lost around 3 weeks after the injection (Fig. 3a). HIP^allo^ grafts injected into the contralateral leg showed stable signals throughout the observation period of 8 weeks or until the scheduled graft recovery. In the group receiving HIP^allo^ first, HIP^allo^ signals remained stable for the entire period of up to 16 weeks (Fig. 3b). The same animals rapidly rejected wt^allo^ grafts in the contralateral leg at 6 weeks, without this immune response affecting the survival of HIP^allo^. Rhesus CD47 was necessary and sufficient to protect the cells from NK cell and macrophage killing, which was observed when allogeneic MHC class I- and II-deficient (DKO) iPSCs were transplanted (Supplementary Fig. 10).

**Fig. 4:**
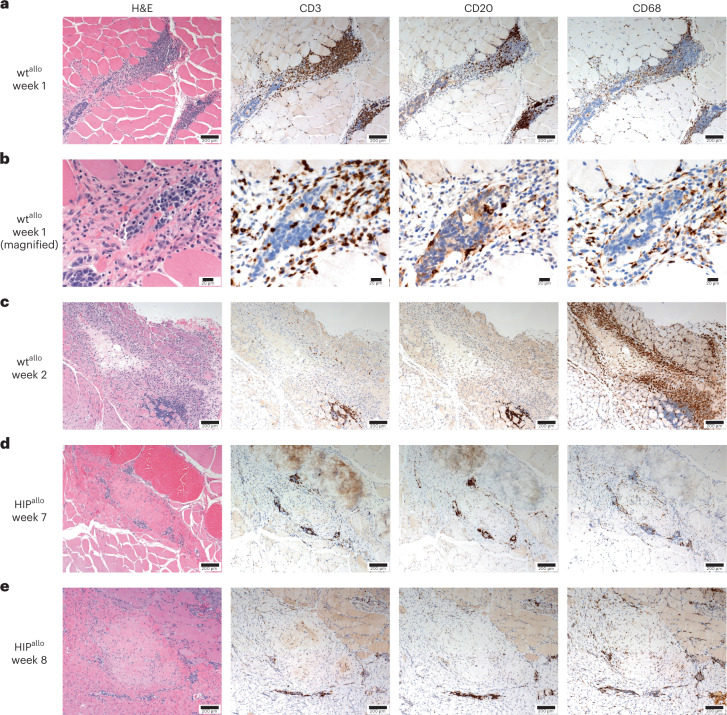
Histology of intramuscular allogeneic rhesus macaque wt and HIP grafts. Representative images of at least two independent sections are shown from H&E staining and immunohistochemical staining for CD3 (T lymphocytes), CD20 (B lymphocytes) and CD68 (macrophages). **a**,**b**, wt^allo^ injection site after 1 week at ×100 (**a**) and ×400 (**b**) magnification. **c**, wt^allo^ injection site after 2 weeks at ×100 magnification. **d**, HIP^allo^ cell injection site after 7 weeks at ×100 magnification. **e**, HIP^allo^ cell injection site after 8 weeks at ×100 magnification.

The wt^allo^ injection sites recovered after 1 week revealed foci of injected cell growth with lineage development consistent with mesodermal and ectodermal lineages (Fig. 4a). The ectodermal lineage consisted of immature neuroectodermal rosettes, whereas the mesodermal lineage had poorly differentiated spindle cells. The early teratomatous mass had abundant mitotic figures and no zones of necrosis and was predominantly located between intact fascicles of muscle with focal interfascicular infiltration. The small mass was associated with intense regional perivascular inflammation that extended into its architecture. Immunohistochemistry demonstrated that the inflammation consisted of B lymphocytes around the regional blood vessels, T lymphocytes around the blood vessels and extending to the graft and increased macrophage infiltrates extending from the blood vessel to the mass. Higher magnification (Fig. 4b) demonstrates that the T lymphocytes and macrophages had entered the teratomatous structures, including the rosettes, and they were associated with early degenerative changes in ectodermal cells closest to the perivascular inflammation. After 2 weeks, the injection site no longer contained identifiable graft cells but, instead, was replaced by an intense collection of macrophages, with a continuing presence of milder perivascular chronic inflammation. Immunohistochemistry (Fig. 4c) demonstrated moderate perivascular B and T lymphocyte collections with a mild interstitial infiltrate of T lymphocytes at the edge of the injection site. Macrophages comprised the vast majority of cells at the injection site, with a substantial number of them radiating out into the nearby muscle fascicles. The HIP^allo^ sites in the contralateral limbs 7 weeks and 8 weeks after injection showed expanding teratomatous masses (Fig. 4d–e). Mitotic figures were present in small numbers, and necrosis was absent within the growths. The cell architectures within the grafts were consistent with differentiation into fibroblasts, smooth muscle cells and osteogenic stroma (with osteoid). Additionally, focal areas of mature cartilage and bone formation were seen in some growths. In contrast to wt^allo^ injection sites, T lymphocytic infiltration into the grafts and perivascular chronic inflammation were greatly reduced. Macrophages were present at low numbers within and around grafts. To better differentiate between immune rejection and unspecific inflammation from teratoma growth, we generated iPSCs from one additional rhesus monkey and injected autologous iPSCs back into the same animal using the same injection technique. After 18 weeks, the autologous iPSCs grew into similar teratomatous masses as HIP^allo^ (Supplementary Fig. 11a–c). There were small numbers of macrophages, but there were perivascular collections of B lymphocytes and T lymphocytes that extended into the teratomas (Supplementary Fig. 11d–g). The immune infiltrate was qualitatively similar to that in HIP^allo^ and no less quantitatively, which supports the notion that HIP^allo^ does not elicit any alloimmune response locally.

### Human HIP cells can be differentiated into pancreatic islet cells

Next, human wt and HIP iPSCs were differentiated into pancreatic islet cells, and, after 22 d, cells showed the typical phenotype of insulin-producing β cells and glucagon^+^ cells (Supplementary Fig. 12a,b) and aggregated into clusters (Supplementary Fig. 12c). The hypoimmune gene edits did not affect the differentiation capacity, and similar amounts of cell types were generated from wt and HIP iPSCs (Supplementary Fig. 12d). We showed previously that human iPSCs and their derivatives express very low levels of NK cell-activating ligands^16^ and that a clear threshold for CD47 overexpression levels can be defined in HIP derivatives that are effective in suppressing NK cell killing^11^. To define such a threshold for human islet cells, primary human islet cells underwent CRISPR–Cas9 inactivation of the *B2M* and *CIITA* genes. These primary islet DKO cells were transduced to express a wide range of CD47 levels by using lentiviral particles in different multiplicities of infection. Twelve cultures of HIP-edited primary islet cells were selected for CD47 quantitation and subsequent NK cell killing assays (Supplementary Fig. 13a). We found a threshold for CD47 expression at approximately 180,000–220,000 per cell, with lower levels leading to killing and higher levels providing protection. Our human HIP iPSCs and the derived islet cells both showed CD47 expression levels of above 600,000 per cell and were, thus, protected from NK cell killing (Supplementary Fig. 13b,c). To correlate CD47 expression, the threshold for the protective HIP phenotype and the virus copy numbers (VCNs) necessary, we performed three more HIP editing runs and included unedited islet cells as well (Supplementary Fig. 13d). We found that CD47 expression levels associated with full innate immune protection were achieved with a VCN of approximately 2 or higher. In diabetic NSG mice, both wt and HIP islets survived and normalized glucose levels with similar efficacies (Supplementary Fig. 14). For subsequent in vivo assays in immunocompetent mice, wt, HLA class I- and II-deficient (DKO) and HIP iPSCs were differentiated into islets. HIP-derived islet cells still showed the hypoimmune phenotype of HLA class I and II depletion and CD47 overexpression (Supplementary Fig. 12e). All three products showed similar insulin release capacities, which were approximately 30% of that of primary unedited human islets (Supplementary Fig. 12f).

### Human HIP islets ameliorate diabetes in immunocompetent allogeneic humanized mice

**Fig. 5:**
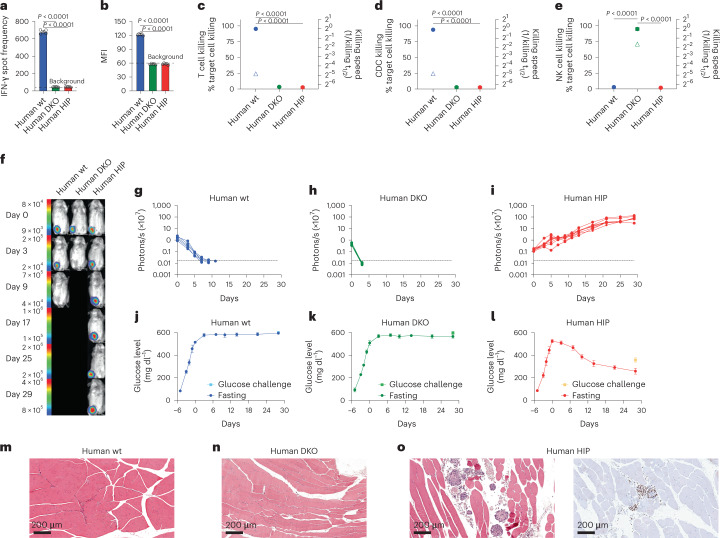
Human HIP islets survive in immunocompetent allogeneic humanized mice and ameliorate diabetes. **a**–**e**, One million wt, DKO or HIP islet cells were injected into the thigh muscle of allogeneic humanized NSG-SGM3 mice. After 6 d, the immune response against the graft cells was assessed. IFN-γ ELISpot assays with mouse splenocytes were performed to assess the adaptive immune response against the islet grafts (**a**, mean ± s.d., three animals per group, one-way ANOVA with Bonferroni post hoc test). Serum DSAs of IgM type were quantified by flow cytometry (**b**, mean ± s.d., three animals per group, one-way ANOVA with Bonferroni post hoc test). Impedance killing assays of islet cells with recipient mouse T cells (**c**, mean ± s.d., three animals per group, one-way ANOVA with Bonferroni post hoc test) or serum (**d**, mean ± s.d., three animals per group, one-way ANOVA with Bonferroni post hoc test). Human NK cells were used for this impedance killing assay of islet cells (**e**, mean ± s.d., triplicates, one-way ANOVA with Bonferroni post hoc test). **f**–**i**, Humanized NSG-SGM3 mice received streptozotocin and developed diabetes (**f**, one representative mouse is shown per group). Six days later, 1,000 FLuc^+^ allogeneic wt (**g**, five animals), DKO (**h**, five animals) or HIP islet clusters (**i**, eight animals) were injected intramuscularly, and their survival was monitored with BLI (all individual animals are shown). **j**–**l**, Serial fasting blood glucose levels were quantified (mean ± s.d.) in the wt (**j**, five animals), DKO (**k**, five animals) or HIP (**l**, eight animals) groups. On day 30, animals underwent a glucose challenge with blood draw 30 min later (mean ± s.d.). **m**–**o**, The injection sites of wt (**m**), DKO (**n**) or HIP islet clusters (**o**) were recovered and stained with H&E and for ISL-1 by immunohistochemistry (representative images).

We assessed the immune response after transplantation of 1 million wt, DKO or HIP islet cells into allogeneic humanized NSG-SGM3 mice. All mice showed more than 45% of human CD45^+^ cells in the peripheral blood, including functionally competent CD3^−^CD7^+^CD56^+^ human NK cells^22^ (Supplementary Fig. 15). After 6 days, there was a strong IFN-γ PBMC response and a surge of IgM DSAs against wt cells but no measurable adaptive immune response against DKO and HIP islet cells (Fig. 5a,b). T cells and serum isolated from recipient mice exerted cytotoxicity against wt islets, and NK cells isolated from human blood killed DKO islets, whereas no response against HIP islets was found (Fig. 5c–e). Next, we assessed survival and glycemic control of 1,000 islet clusters in diabetic allogeneic humanized NSG-SGM3 mice. BLI showed rapid vanishing of wt and DKO islets, whereas HIP islets survived and improved in metabolic function over time (Fig. 5f–i). Both wt and DKO islet transplants showed no effect on glucose levels (Fig. 5j,k). HIP islets, however, steadily reduced fasting hyperglycemia and approached the 200 mg dl^−1^ level after 28 d (Fig. 5l). Only HIP islets kept glucose levels below 400 mg dl^−1^ after glucose challenge. Histology of the injection sites in the thigh muscles after 28 d for wt and DKO islet clusters showed no evidence of persistent injected cells, and there was not any apparent inflammation remaining (Fig. 5m,n). In contrast, HIP injection sites had well-formed islets without inflammation (Fig. 5o). The islets consisted of cohesive clusters of monomorphic cuboidal cells with central nuclei with finely granular chromatin. The nuclei stained positively for insulin gene enhancer protein (ISL-1) by immunohistochemistry, as would be expected for well-differentiated endocrine islets. Surrounding the islets were occasional collections of necrotic endocrine cells, most likely due to the cell transplantation procedure given the consistent absence of immune cells.

### Allogeneic HIP islets achieve long-term survival in rhesus macaques

**Fig. 6:**
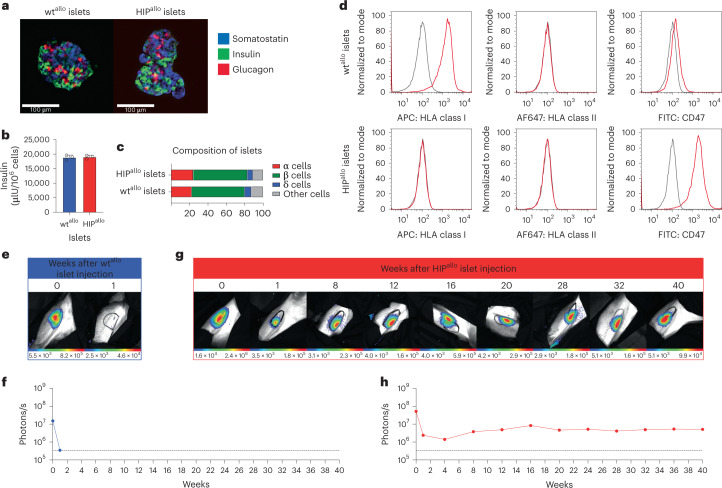
Allogeneic HIP islets achieve long-term survival. **a**, Immunofluorescence pictures of dissociated and reaggregated wt^allo^ and HIP^allo^ islets from rhesus monkeys (representative images). **b**, In vitro insulin secretion of wt^allo^ and HIP^allo^ islets (mean ± s.d., triplicates). **c**, The composition of wt^allo^ and HIP^allo^ islets of α, β, δ and other cells (mean, triplicates). **d**, Flow cytometry histograms for HLA class I and II and rhesus CD47 on wt^allo^ and HIP^allo^ islets (representative images). **e**–**h**, The survival of FLuc^+^ wt^allo^ and HIP^allo^ islets in allogeneic rhesus monkeys was followed by BLI (one animal each).

All attempts to generate β cells from non-human primate iPSCs so far have failed to meet minimum requirements for phenotype and function. To still test the survival of HIP^allo^ islets in primates without the need for iPSC differentiation, we performed HIP editing on primary rhesus macaque islets. The control wt islets underwent the same protocol just without the editing. Both wt^allo^ and HIP^allo^ islets showed similar morphology, function and cell composition (Fig. 6a–c). HIP^allo^ islets demonstrated the HIP phenotype (Fig. 6d). FLuc^+^ wt^allo^ and HIP^allo^ islets were transplanted into one allogeneic rhesus macaque each. Whereas wt^allo^ islets were rejected within 1 week, HIP^allo^ islets achieved long-term survival with stable BLI signals over 40 weeks (Fig. 6e–h). Immune analyses showed a strong alloresponse with cellular and antibody-mediated islet cell killing in the animal that received wt^allo^ islets (Supplementary Fig. 16) and a complete lack of immune response after HIP^allo^ islet transplantation (Supplementary Fig. 17).

## Discussion

Hypoimmune engineering of cells aims to avoid immune recognition by innate and adaptive immune cells. With HLA-G or HLA-E engineering, cells retain the ability to present a repertoire of peptides^23,24^, and we saw some activation of adaptive immunity by transduced K562 in humanized mice. The inclusion of a linked non-polymorphic peptide into the HLA-E construct^14^ might mitigate such T cell interactions. However, we could confirm that only subpopulations of NK cells express the inhibitory receptors ILT2 and CD94/NKG2A for HLA-G and HLA-E^25^, respectively, and we could show that K562^HLA-G^ and K562^HLA-E^ remained susceptible to NK cell killing by effector cells lacking such inhibitory signals. PD-1 is a major immune checkpoint in T cells, but because very few NK cells express PD-1 (ref. ^26^), this immune checkpoint is insufficient to inhibit innate immunity. In the K562 model used herein, only transgenic CD47 overexpression provided widespread protection against unsorted human peripheral NK cells.

The rhesus macaque immune system largely resembles that of humans, but awareness of differences in marker expression of immune cells^27,28^, incompatibility of immune regulatory pathways^11^, species-specific xenoantigens^29,30^ and the special role of inflammation in xenotransplantation^31^ is critical when designing and evaluating rhesus macaque immune studies. Therefore, we chose to overexpress rhesus CD47 in human HIP^xeno^ and rhesus macaque HIP^allo^ to assure compatibility with its SIRPα receptor on rhesus macaque immune cells. The functionality of the interaction between CD47 and SIRPα was shown previously^11^, and we did not measure local SIRPα expression in this study. We observed pre-formed anti-human antibodies binding to wt^xeno^ but not HIP^xeno^, thus most likely recognizing HLA epitopes. Despite the complexity of immune interactions involved in xenotransplantation, complete systemic immune evasion was achieved with our hypoimmune concept.

In the allogeneic setting, HIP^allo^ did not induce any measurable immune cell activation and were not susceptible to the vigorous cellular and antibody-mediated cytotoxicity incited by concomitantly transplanted wt^allo^. The ability to evade immune rejection even in previously wt^allo^-injected and sensitized recipients could enable repeat dosing of a potential future HIP-engineered cell product. Notably, our HIP^allo^ implants reliably showed survival in fully allogeneic rhesus macaque recipients and gave rise to different tissues of different germ layers. A previous study showed that teratoma formation from rhesus macaque iPSCs was less efficient in autologous monkeys than in NSG mice, and only a fraction of iPSC implants in autologous monkeys developed into tumors^32^. This suggests that the survival of HIP^allo^ is at least equivalent to that of autologous iPSCs. The fact that HIP^allo^ gave rise to a variety of different cell types, which collectively avoided immune recognition and achieved survival and engraftment in immunocompetent allogeneic rhesus macaques, supports the notion that the hypoimmune features are maintained during differentiation and can protect various HIP^allo^-derivatives. This concept could be confirmed with HIP-edited primary rhesus monkey islets, which achieved long-term survival in an allogeneic recipient.

In vivo differentiation into the three germ layers did not occur equally, and we observed a tendency to differentiate into mesodermal lineages. There is evidence that differentiation is affected by environmental cues, such as growth factors^33,34^ and extracellular matrix^35,36^, as well as electrical and mechanical stimuli^37^, which direct differentiation and maturation. We, therefore, found a dominance of developing muscle, cartilage and bone at the HIP^allo^ implant sites in quadriceps muscles. The latter two tissues, however, dampen the BLI signal of underlying cells, and we would expect that further increase in ossification would jeopardize our ability to quantitatively assess cell survival using BLI.

The histology of the wt^allo^ injection areas showed typical features of allograft rejection with intense early T lymphocyte and macrophage infiltrations, followed by a phase of macrophage phagocytosis and clearance of graft cells. In contrast, the histology of the HIP^allo^ injection sites showed growing teratomatous structures with much lower levels of immune cell infiltration. There was a persistence of mild perivascular chronic inflammation in all grafts, consisting of a mixture of B and T lymphocytes with small numbers of plasma cells. The same pattern was seen after autologous iPSC transplantation and was consistent with chronic non-specific inflammation that is found in a broad range of pathology types in which there is a physical etiology of tissue irritation, such as tensile forces or expansile masses. Together, our results suggest that HIP^allo^ injection does not cause any local allorejection response.

We showed previously that human HIP can be differentiated into endothelial cells and cardiomyocytes^16^, and we now show that HIP can differentiate into pancreatic islet cells that survive and ameliorate diabetes in immunocompetent allogeneic humanized mice. This supports the prospect of manufacturing human HIP-derived islet cells for therapeutic use in the future. Furthermore, the long-term survival of HIP-edited primary rhesus macaque islets supports the notion that immune-engineered islet cells can persist in allogeneic recipients without any immunosuppression. Engineered HIP-derived islet cell therapeutics thus hold great promise to achieving long-term survival and glycemic control in patients with diabetes.

## Methods

### Human K562 experiments

#### K562 cell culture and transduction of HLA-E, HLA-G, PD-L1 and CD47 transgenes

K562 cells were purchased from the American Type Culture Collection and cultured in RPMI 1640 plus 10% FCS hi (Gibco) and 1% penicillin–streptomycin. For the lentiviral transduction, cells were plated in six-well plates at a density of 1 × 10^5^ cells per well per 2 ml of growth medium containing protamine sulfate (500 μg ml^−1^). Spinfection was carried out at 135*g* for 10 min at 25 °C. A multiplicity of infection (MOI) of 10 was used for lentiviral particles carrying cDNA transgenes for HLA-E, HLA-G, PD-L1 and CD47 (all Thermo Fisher Scientific). After 24 h, virus was removed, and complete media change was performed. Cells underwent sorting using anti-HLA-E (APC, clone 3D12, 342605, 1:20 dilution, BioLegend with IgG1, APC, clone MOPC-21, 400121 1:20 dilution, BioLegend), anti-HLA-G (APC, clone 87G, 335909, 1:20 dilution, BioLegend with IgG2a, APC, clone MOPC-173, 400221 1:20 dilution, BioLegend), anti-PD-L1 (PE, clone 29E.2A3, 329705, 1:20 dilution, BioLegend with IgG2b, PE, clone MPC-11, 400313, 1:20 dilution, BioLegend) or anti-CD47 (FITC, clone CC2C6, 323106, 1:20 dilution, BioLegend with IgG1, FITC, clone MOPC-21, 400110, 1:20 dilution, BioLegend) using a FACSAria Fusion. Representative plots are shown.

#### NK cell culture and sorting

Human primary peripheral blood NK cells were purchased from STEMCELL Technologies (70036) and stimulated with 1 μg ml^−1^ human IL-2 (PeproTech) overnight in RPMI 1640 plus 10% FCS hi and 1% penicillin–streptomycin. Cells were stained and sorted using anti-CD56 (PerCP/Cy5.5, clone MEM-188, 304625, 1:20 dilution, BioLegend with IgG2a, PerCP/Cy5.5, clone MOPC-173, 400251, 1:20 dilution, BioLegend), anti-SIRPα (APC, clone 15-414, 372105, 1:20 dilution, BioLegend with IgG2a, APC, clone MOPC-173, 400219, 1:20 dilution, BioLegend), anti-CD94 (FITC, clone DX22, 305504, 1:20 dilution, BioLegend with IgG1, FITC, clone MOPC-21, 400107, 1:20 dilution, BioLegend), anti-CD3 (AF488, clone UCHT1, 300415, 1:20 dilution, BioLegend with IgG1, AF488, clone MOPC-21, 400129, 1:20 dilution, BioLegend), anti-ILT2 (APC, clone GHI/75, 333719, 1:20 dilution, BioLegend with IgG2b, APC, clone MPC-11, 400321, 1:20 dilution, BioLegend) and anti-PD-1 (FITC, clone EH12.2H7, 329903, 1:20 dilution, BioLegend with IgG1, FITC, clone MOPC-21, 400107, 1:20 dilution, BioLegend). The sorted population (FACSAria Fusion with FACSDiva 8.0.3, BD Biosciences) was used as effector cells.

#### NK cell killing by xCELLigence

NK cell killing assays were performed on the xCELLigence MP platform (ACEA Biosciences). Specialized 96-well E-plates (ACEA Biosciences) were coated with tumor-coating solution (Agilent), and 4 × 10^4^ target K562 were plated in 100 μl of media. After the cell index reached 0.7, the effector cells were added at an effector cell to target cell (E:T) ratio of 1:1. NK cells were stimulated with 1 μg ml^−1^ human IL-2 (PeproTech). As killing control, cells were treated with 2% Triton X-100 in water. Data were standardized and analyzed with RTCA 2.1 software (ACEA Biosciences). Supernatants were collected for granzyme B and perforin ELISA after 90 h.

#### Granzyme B and perforin ELISA

Supernatants of NK cell killing in xCELLigence were collected and analyzed for granzyme B and perforin (both Thermo Fisher Scientific) in ELISA assays according to the manufacturerʼs instructions. Samples were analyzed in a microplate reader using Kaleido 3.0 software (Thermo Fisher Scientific).

#### Mice

Male NSG mice (strain 005557) and female humanized NSG-SGM3 mice (strain 013062) were purchased from The Jackson Laboratory and used as recipients for different assays, as described previously^16^. Humanized mice were not thymectomized, received human CD34^+^ cells at 12 weeks of age and were included into study groups 6–8 weeks after humanization. Animals were randomly assigned to experimental groups. Some mice received 200 μl of clodronate (LIPOSOMA, stock concentration 5 mg ml^−1^) intraperitoneally 3 d before cell injection. The number of animals per experimental group is presented in each figure. Mice were housed in 12-h light/dark cycles with humidity between 30% and 70% at ambient temperature of 20–26 °C. The study and control animals were housed in the same room. The animal facility is a specific pathogen-free facility.

#### ELISpot

For uni-directional ELISpot assays, recipient splenocytes were isolated 6 d after the humanized mice received the cell injections, and splenocytes were plated as recipient cells. Donor cells were treated with mitomycin (50 μg ml^−1^ for 30 min, Sigma-Aldrich) and used as stimulator cells. A total of 5 × 10^5^ stimulator cells were incubated with 5 × 10^5^ recipient responder PBMCs for 24 h, and IFN-γ spot frequencies (BD Biosciences) were enumerated using an ELISpot plate reader (AID Diagnostika).

#### DSA

Sera from recipient mice were de-complemented by heating to 56 °C for 30 min. Equal amounts of sera and cell suspensions (5 × 10^6^ per milliliter) were incubated for 45 min at 4 °C. Cells were labeled with FITC-conjugated goat anti-human IgM (Thermo Fisher Scientific), and mean fluorescence intensity (MFI) was analyzed by flow cytometry (Attune, Thermo Fisher Scientific).

#### Mouse in vivo innate cytotoxicity assay

Five million human T cells and 5 million K562, K562^HLA-E^, K562^HLA-G^, K562^PD-L1^ or K562^CD47^ were mixed and injected intraperitoneally into NSG mice. T cells were labeled with DiO, and K562 cells were labeled with DiD according to the manufacturer’s protocol (Vybrant Multicolor Cell-Labeling Kit, Invitrogen). After 48 h, cells were collected from the abdomen and analyzed for the percentages of DiO^+^ and DiD^+^ cells by flow cytometry (Attune). All animals were pre-treated 18 h before the assay with poly I:C injection (100 μg in sterile PBS, intraperitoneal injection, Sigma-Aldrich).

#### Human iPSCs

##### Generation of human HIP^xeno^

The Human Episomal iPSC Line was purchased from Thermo Fisher Scientific. Human HIP^xeno^ were generated from wt^xeno^, as described previously^11,16^.

##### Human iPSC culture, transduction to express FLuc and flow cytometry

Human iPSCs (wt^xeno^ and HIP^xeno^) were cultured in Essential 8 Flex media (Thermo Fisher Scientific) on 10-cm dishes coated with diluted feeder-free Matrigel (hESC qualified, BD Biosciences). Media was changed every 24 h, and VERSENE (Gibco) was used for cell passaging with a ratio of 1:6. For FLuc transduction, 10^5^ iPSCs were plated in one six-well plate and incubated overnight at 37 °C at 5% CO_2_. The next day, media was changed, and one vial of Fluc lentiviral particles expressing luciferase II gene under re-engineered EF1a promotor (Gentarget) was added to 1.5 ml of media. After 36 h, 1 ml of cell media was added, and, after another 24 h, complete media change was performed. Two days later, luciferase expression was confirmed by adding d-luciferin (Promega). Signals were quantified in units of maximum photons per second per centimeter square per steradian (p/s/cm^2^/sr). To assess HLA class I expression, cells were harvested and labeled with APC-conjugated anti-HLA-A,B,C antibody (clone G46_2.6, 555555, 1:20 dilution, BD Biosciences) or APC-conjugated IgG1 isotype-matched control antibody (clone MOPC-21, 554681, 1:5 dilution, BD Biosciences). To assess HLA class II expression, cells were incubated with Alexa Fluor 647-labeled anti-HLA-DR,DP,DQ antibody (clone Tu39, 563591, 1:20 dilution, BD Biosciences) or Alexa Fluor 647-labeled IgG2a isotype-matched control antibody (clone G155-178, 565357, 1:60 dilution, BD Biosciences). To assess CD47 expression, the PerCP-Cy5-conjugated anti-CD47 (clone B6H12, 561261, 1:20 dilution, BD Biosciences) or PerCP-Cy5-conjugated IgG1 isotype-matched control antibody (clone MOPC-21, 550795, 1:20 dilution, BD Biosciences) was used. Representative histograms acquired on the Attune NTx flow cytometer are shown. Gating strategies are provided in Supplementary Fig. 18.

##### Quantification of CD47 expression

Human primary islet cells, iPSCs and iPSC-derived islet cells were stained with Zombie NIR Fixable Viability Kit (77184, BioLegend), and Fc block (422302, BioLegend) was added on ice for 10 min. PE-labeled anti-CD47 antibody (clone CC2C6, 323108, 1:20 dilution, BioLegend) was used to stain the cells for 30 min on ice and then washed twice in FACS buffer. Then, 500 μl of PBS was added to Quantibrite beads (340495, BD Biosciences) and acquired on the Attune flow cytometer. Analysis was performed according to the manufacturer’s instructions on FlowJo 10 software. Gating strategies are provided in Supplementary Fig. 18.

##### Islet cell differentiation of human wt and HIP

The differentiation was adapted from the previously reported protocol^38,39^ with the differences that no antibiotics were used.

##### Imaging of islet cell clusters

Approximately 20 μl of aggregates was collected into a 1.5-ml Eppendorf tube, washed with DPBS, fixed in 4% paraformaldehyde (PFA) in DPBS for 1 h at 4 °C, washed with DPBS and stored in DPBS at 4 °C until staining (<1 week). Aggregates were incubated in 100% methanol pre-cooled at −20 °C for 30 min, and then 900 μl of methanol was replaced with 900 μl of DPBS. Samples were spun down and washed twice with DPBS. Aggregates were incubated in penetration buffer (0.2% Triton X-100, 2.5% BSA, 20% DMSO, DPBS) for 1 h at room temperature on an orbital shaker (240 r.p.m.), followed by 1-h incubation in blocking buffer (0.2% Triton X-100, 2.5% BSA, 10% DMSO, DPBS). Then, 500 μl of aggregates in buffer was transferred to a new tube, and the buffer was replaced with 300 μl of primary antibodies (rat anti-C-peptide, polyclonal, MAB14171, 1:20 dilution, Novus Biologicals; mouse anti-NKX6.1, polyclonal, NBP1-49672, 1:20 dilution, Novus Biologicals; and rabbit anti-CHGA, polyclonal, NB120-15160, 1:20 dilution, Novus Biologicals) in antibody buffer (0.2% Triton X-100, 2.5% BSA, 5% DMSO, DPBS) and incubated for 24 h at room temperature on an orbital shaker. The samples were washed five times with 0.2% Triton in DPBS and then incubated in secondary antibodies (AF488-conjugated donkey anti-rat Ig, polyclonal, A-11006, 1:500 dilution, Thermo Fisher Scientific; AF647-conjugated donkey anti-mouse Ig, polyclonal, A-21235, 1:500 dilution, Thermo Fisher Scientific; and AF647-conjugated donkey anti-rabbit Ig, polyclonal, A-21244, 1:500 dilution, Thermo Fisher Scientific) in antibody buffer for 24 h at room temperature on an orbital shaker. Aggregates were washed five times with 0.2% Triton in DPBS and then resuspended in 250 μl of 60% methanol and incubated for 5 min. Then, 250 μl of 100% methanol was added and incubated for an additional 5 min. Samples were washed with 100% methanol and incubated for 30 min. Methanol was replaced with BABB (1:2 ratio of benzyl alcohol:benzyl benzoate), and samples were mixed using a wide-bore pipette and incubated for 10 min. Aggregates were mounted (90% glycerol, DPBS, 10 mM Tris-HCl) on slides and imaged on an LSM 880 upright confocal microscope (Zeiss) and analyzed with ZEN Blue/Black 2012 image processing software (linear Gaussian filter, maximum intensity projection).

##### Histology of transplanted islet cell clusters

Muscle sections were cut at 4 µm for histological analysis in hematoxylin and eosin (H&E) staining. Islets were identified using an anti-islet 1 antibody (clone EPR10362, ab178400, 1:250 dilution, Abcam) with human pancreas used as a positive control. Blocking was done with 1× Animal-Free Blocker (SP-5030-250, Thermo Fisher Scientific) in 2% NGS (5425S, Cell Signaling Technology) diluted in TBST (J77500.K2, Thermo Fisher Scientific). The primary antibody was diluted in blocking solution at a titer of 1:1,000. Heat-mediated antigen retrieval was done on the Leica BOND using EDTA-based pH 9 solution (AR9640, Leica). A Leica BOND detection kit (DS9800) was used (post-primary step was omitted) for DAB chromogenic staining.

##### Quantification of human insulin

One hundred islet clusters were plated in one six-well plate and 2 ml of islet media with 5.8 mM glucose (PIM(S) media, Prodo). Supernatants were collected after 24 h, and ELISA assay for human insulin (KAQ1251, Thermo Fisher Scientific) was performed according to the manufacturer’s protocol. In brief, islet cell supernatant was incubated with an anti-insulin antibody, followed by incubation with a horseradish peroxidase (HRP)-conjugated secondary antibody and a peroxidase substrate. A microplate reader with an absorbance of optical density (OD) of 450 nm (Molecular Devices) was used to measure the insulin level of the standards and study samples. Insulin levels were calculated as µlU per million cells.

##### FLuc transduction of hiPSC-derived islet cells

iPSC-derived islets were transduced to express FLuc. Five hundred islet clusters were plated in one six-well plate and transduced with FLuc lentiviral particles expressing luciferase II gene under re-engineered CAG promotor (Gentarget) with an MOI of 20. After 36 h, 1 ml of islet cell media was added. After a further 24 h, complete media change was performed. After 2 d, luciferase expression was confirmed by adding d-luciferin (Biosynth). Signals were quantified with Ami HT (Spectral Instruments Imaging) in maximum p/s/cm^2^/sr.

##### Induction and diabetes and transplantation of iPSC-derived islet clusters

To induce diabetes, animals received daily intraperitoneal injection of streptozotocin for five consecutive days at 60 mg kg^−1^ body weight in 0.1 M citrate buffer. Glucose levels were determined by glucometer (Accu-Chek Advantage, Roche) in 10 μl of blood samples collected by tail vein venipuncture after 4 h of fasting. Animals with a glucose level of more than 450 mg dl^−1^ were served as recipients for cell implant. One thousand islet clusters were resuspended in 60 μl of sterile saline and injected intramuscularly into the hindlimb muscle with a 23-gauge needle. Glucose tolerance testing was performed at study endpoint (30 d after cell transplantation). Mice were fasted for 4 h, and a baseline blood glucose level was obtained. A glucose bolus of 2 g kg^−1^ glucose was injected intraperitoneally, and blood glucose levels were measured at 30 min after injection. Blood was collected from study animals, and plasma was stored at −80 °C for ELISA C-peptide (Mercodia) according to the manufacturer’s protocol.

##### Isolation of human NK cells from humanized NSG-SGM3 mice for xCELLigence

The following antibodies were used to characterize the immune cell populations in humanized mice: anti-human CD45 (PE, clone HI30, 304008, 1:20 dilution, BioLegend with IgG1, PE, clone MOPC-21, 400140 1:20 dilution, BioLegend); antiCD19 (PerCP, clone HIB19, 302228, 1:20 dilution, BioLegend with IgG1, PerCP, clone MOPC-21, 400148 1:20 dilution, BioLegend); anti-CD3 (AF488, clone UCHT1, 300415, 1:20 dilution, BioLegend with IgG1, AF488, clone MOPC-21, 400129 1:20 dilution, BioLegend); anti-CD33 (BV605, clone P67.6, 366612, 1:20 dilution, BioLegend with IgG1, BV605, clone MOPC-21, 400162 1:20 dilution, BioLegend); anti-CD7 (APC, clone 4H9/CD7, 395605, 1:20 dilution, BioLegend with IgG2a, APC, clone MOPC-173, 400219, 1:20 dilution, BioLegend); anti-CD56 (PerCP/Cy5.5, clone MEM-188, 304625, 1:20 dilution, BioLegend with IgG2a, PerCP/Cy5.5, clone MOPC-173, 400251, 1:20 dilution, BioLegend); anti-F4/80 (APC, clone QA17A29, 157305, 1:40 dilution, BioLegend with IgG1, APC, clone MOPC-21, 400119, 1:20 dilution, BioLegend); anti-CD68 (FITC, clone Y1/82A, 333805, 1:20 dilution, BioLegend with IgG2b, FITC, clone MPC-11, 400309, 1:20 dilution, BioLegend); and anti-mouse CD45 (AF700, clone I3/2.3, 147715, 1:50 dilution, BioLegend with IgG1, AF700, clone RTK4530, 400628, 1:20 dilution, BioLegend).

Splenocytes were isolated from humanized mice and sorted for the CD3^−^ (AF488, clone UCHT1, 300415, 1:20 dilution, BioLegend), CD7^+^ (APC, clone 4H9/CD7, 395605, 1:20 dilution, BioLegend) and CD56^+^ (PerCP/Cy5.5, clone MEM-188, 304625, 1:20 dilution, BioLegend) NK cell populations. Sorted NK cells were cultured and activated overnight in RPMI 1640 + 10% FCS hi and 1% penicillin–streptomycin. Killing assays of human DKO iPSCs (4 × 10^4^ cells in 100 μl of media) with these NK cells were performed on the xCELLigence MP platform. After the cell index reached 0.7, the NK cells were added at an E:T ratio of 1:1. NK cells were stimulated with 1 μg ml^−1^ human IL-2 (PeproTech). As killing control, cells were treated with 2% Triton X-100 in water. Data were standardized and analyzed with RTCA 2.1 software (ACEA Biosciences). Measurement was performed every 2 min for 6 h and then every 15 min for a total of 48 h.

##### DNA extraction and digital droplet PCR methods for VCN

Genomic DNA (gDNA) was extracted using Lucigen QuickExtract according to the manufacturerʼs recommendations. The gDNA stock was then diluted at three-point two-fold dilutions using ultra-pure nuclease-free water, and 5 μl of each dilution will be used per reaction in triplicates. VCN was measured by digital droplet PCR (ddPCR) using a FAM-conjugated primer probe targeting the DelU3 region and a HEX-conjugated primer probe targeting ARX, used as a reference gene. The ddPCR reaction was set up in a 96-well plate format according to the manufacturerʼs recommendations using the Bio-Rad ddPCR Multiplex Supermix, primers (900 nM), probes (250 nM) and gDNA in a final reaction volume of 22 μl. The reaction plate was sealed with adhesive foil seals (Bio-Rad), vortexed vigorously and then spun down at 135*g* for 1 min using a tabletop centrifuge. The ddPCR reaction plate was then transferred to the Automated Droplet Generator, and the manufacturerʼs protocol was followed to generate droplets. The sample droplet plate was then sealed using the recommended foil and sealer and then transferred onto a C1000 Touch thermal cycler (Bio-Rad) where droplets were amplified to endpoint by heating to 95 °C for 10 min, followed by 40 cycles of 94 °C for 30 s and 60 °C for 2 min (using ramp rate of 2 °C s^−1^), with a final heating step of 98 °C for 10 min. The reacted products were held at 4 °C. The plate was then placed into the QX200 droplet reader. Using Bio-Rad’s QX Manager Software (Standard Edition, version 1.2), the concentration of the target amplicon per microliter for each sample was estimated for both DelU3 and ARX reference genes. Estimated VCN values were finally calculated by dividing the concentration of the target copies per microliter by the concentration of the reference copies per microliter and multiplied by copy number of reference (VCN = copies DelU3 per µl / copies ARX per µl × 1).

**Table:** 

Name	Sequence
DelU3 forward primer	5′-GGAAGGGCTAATTCACTCCC-3′
DelU3 reverse primer	5′-GGTTTCCCTTTCGCTTTCAGG-3′
DelU3 probe	5′-56-FAM-TGCCCGTCTGTTGTGTGACTCTG-3′-IBFQ
ARX forward primer	5′-TATGTTCAGATG CCCATTAGGG-3′
ARX reverse primer	5′-CTTGCTCAAAGGACTGTGATTTC-3′
ARX probe	5′-HEX-AGTGCCTTTCAGATGGAAACGGGT-3′-IBFQ

### Rhesus macaque iPSCs

#### Generation of rhesus macaque HIP^allo^

The starting rhesus macaque iPSC line ZH26-H16 (wt^allo^) was kindly provided by the National Institutes of Health. The line was characterized by karyotyping (also known as G-banding, performed under contract by Diagnostic Cytogenetics), which indicated that the line had a normal complement of chromosomes, is genetically male and did not harbor any clonal chromosomal breaks. Ablation of the MHC class I and II proteins was achieved by targeted cleavage of the *B2M* and *CIITA* genes, respectively, using CRISPR–Cas9 genome editing. The guides CGUGAGUAAACCUGAAUCUU and GAUAUUGGCAUAAGCCUCCC were used to target *B2M* and *CIITA*, respectively. Single cells of the *B2M*^*−/−*^*CIITA*^*−/−*^ ZH26-H16 bulk were printed into 96-well plates using the Namocell Hana printer. Single cells were expanded over 10 d and monitored for single-cell colony outgrowth. Two clones (1-B4 and 2-B5) were selected for subsequent CD47 transduction. A lentiviral transfer vector was designed to encode rhesus CD47 in an expression cassette driven by the CAG promoter. The complete annotated sequence of the CD47 transgene was as we described previously^11^. The resulting pool of cells was characterized by flow cytometry.

#### Rhesus macaque iPSC culture and flow cytometry

Rhesus wt^allo^ and HIP^allo^ were cultured on diluted laminin-coated (Thermo Fisher Scientific) T75 flasks in PluriSTEM Media (MilliporeSigma). Medium was changed every 24 h, and Accutase was used for cell passaging at a ratio of 1:8 to 1:10 every 3 d. RevitaCell supplement was added for the first 24 h. FLuc transduction was performed as outlined above for human cells. Cells were harvested and labeled with APC-conjugated anti-HLA-A,B,C antibody (clone G46_2.6, 555555, 1:20 dilution, BD Biosciences; clone G46_2.6 has shown cross-reactivity with rhesus macaque MHC class I) or APC-conjugated IgG1 isotype-matched control antibody (clone MOPC-21, 554681, 1:5 dilution, BD Biosciences). Cells were labeled with Alexa Fluor 647-conjugated anti-HLA-DR,DP,DQ antibody (clone Tu39, 563591, 1:20 dilution, BD Biosciences; clone Tu39 has shown cross-reactivity with rhesus macaque MHC class II) or Alexa Fluor 647-conjugated IgG2a isotype-matched control antibody (clone G155-178, 565357, 1:60 dilution, BD Biosciences). Also, cells were labeled with FITC-conjugated anti-CD47 antibody (clone CC2C6, 323106, 1:20 dilution, BioLegend) or FITC-conjugated mouse IgG1k isotype-matched control antibody (clone MOPC-21, 400110, 1:20 dilution, BioLegend). Representative histograms are shown. Gating strategies are provided in Supplementary Fig. 18.

### Primary rhesus macaque islet experiments

#### Gene editing of primary rhesus macaque islets

Primary rhesus macaque cadaveric islets were a kind gift from the Kievit Laboratory (Oregon Health & Science University). CRIPSR–Cas9 technology was used for the disruption of the *B2M* and *CIITA* genes. Islet clusters were dissociated in single cells using Accumax (STEMCELL Technologies) for 10 min at 37 °C. The following gRNA sequences were used for the macaca mulatta *B2M* gene 5′-CGUGAGUAAACCUGAAUCUU-3′ and macaca mulatta *CIITA* gene 5′-GAUAUUGGCAUAAGCCUCCC-3′. Lonza P3 Primary Cell 4D-Nucleofector X Kit (V4XP-3032) was used for the transfection of the islet cells. In brief, cells were transduced with a final concentration of 50 million per milliliter in P3 buffer. The cell suspensions (20 μl) were pipetted in one well of the eight-strip containing 13 μg of Cas9 enzyme and 5 μM single guide RNA, respectively. Lonza’s 4D-Nucleofector was used for the electroporation with the preset program CA-137. Islet cells were transferred in U-bottom 96-well plates containing 50,000 cells per well in PIM(S) media (Prodo) and rested for 1 h at 37 °C and 5% CO_2_ before moving the plate on the Belly Dancer orbital shaker (IBI Scientific) for islet re-clustering. Complete media change was performed after 48 h, and islet clusters were incubated on the Belly Dancer for another 24 h. Islet clusters were dissociated again in single cells using Accumax for cell sorting using the anti-HLA-A,B,C antibody (clone G46_2.6, BD Biosciences) or IgG1 isotype-matched control antibody (clone MOPC-21, BD Biosciences) and anti-HLA-DR,DP,DQ antibody (clone Tu3a, BD Biosciences) or IgG2a isotype-matched control antibody (clone G155-178, BD Biosciences). Double-negative cells were sorted in the BD FACSAria II and replated in U-bottom 96-well plates as described above for islet re-clustering on the Belly Dancer orbital shaker. After 24 h, islets were dissociated in single cells for rhesus CD47 and luciferase transduction with a CAG-CD47 LVV (custom order, Thermo Fisher Scientific) at an MOI of 5 and a CAG-luciferase LVV (custom order, Gentarget) at an MOI of 20. Spinfection was performed with the presence of 10 μg ml^−1^ protamine sulfate at 300*g* for 15 min. Cells were replated in U-bottom 96-well plates as described above for islet re-clustering on the Belly Dancer orbital shaker. After 48 h, cells were dissociated in single cells using Accumax and underwent cell sorting for rhesus CD47 with anti-CD47 antibody (clone CC2C6, BD Biosciences) or IgG1 isotype-matched control antibody (clone MOPC-21, BD Biosciences) on a BD FACSAria II. Luciferase expression was confirmed by adding d-luciferin (Promega). Signals were quantified with Ami HT (Spectral Instruments Imaging) in maximum p/s/cm^2^/sr. Islet cells were replated in U-bottom 96-well plates as described above for islet re-clustering on the Belly Dancer orbital shaker until transplantation.

#### Quantification of islet cluster insulin production

One hundred islet clusters were plated in one six-well plate and 2 ml of islet media with 5.8 mM glucose (PIM(S) media, Prodo). Supernatants were collected after 24 h, and ELISA assays for rhesus insulin (MBS701773, MyBiosource) were performed according to the manufacturer’s protocol. A microplate reader with an absorbance of OD 450 nm (Molecular Devices) was used to measure the insulin level of the standards and study samples. Insulin levels were calculated as µlU per million cells.

#### Quantification of islet composition

Rhesus macaque islets were dissociated with Accumax (STEMCELL Technologies) into single cells. The following antibodies were used for determining islet composition: anti-insulin antibody (AF647, clone 2D11-H5, sc-8033 AF647, dilution 1:20, Santa Cruz Biotechnology with IgG1, AF647, clone MOPC-21, 400130, 1:20 dilution, BioLegend), anti-glucagon antibody (PE, clone C-11, sc-514592 PE, dilution 1:20, Santa Cruz Biotechnology with IgG1, PE, clone MOPC-21, 400113, 1:20 dilution, BioLegend) and anti-somatostatin antibody (FITC, clone G-10, sc-55565 FITC, dilution 1:20, Santa Cruz Biotechnology with IgG2b, PE, clone MPC-11, 400309, 1:20 dilution, BioLegend). Samples were measured on a flow cytometer. Results are shown as percentage of unmodified wt islets or percentage of HIP islets.

### Rhesus macaque transplant experiments

#### Rhesus macaques and procedures

All rhesus macaque experiments were approved by the Alpha Genesis Institutional Animal Care and Use Committee and regulated by the US Department of Agriculture. Six female and 17 male rhesus macaques (3–4 kg) were used. Anesthesia was performed using ketamine (10–20 mg kg^−1^), tiletamine and zolazepam (5–8 mg kg^−1^) and isoflurane (1–4%).

#### Blood collection

Blood was collected from the femoral vein using a 22-gauge needle, a vacutainer sheath and a collection tube. After venipuncture, manual compression of the vein was maintained until hemostasis was achieved. Blood collection days are indicated in the figures and are based on the weight of the animals not exceeding AGI maximum bleeds as set forth by the Institutional Animal Care and Use Committee.

#### Preparation of human wt^xeno^ and HIP^xeno^ iPSCs and injection into rhesus macaques

Human wt^xeno^ and HIP^xeno^ iPSCs were resuspended in 1 ml of diluted high-concentration Matrigel (Corning) including pro-survival cocktail (200 μM ZVAD and 100 nM BcL-xL (both Millipore)), 200 ng ml^−1^ IGF-1 (PeproTech), 100 μM pinacidil and 200 nM cyclosporine A (both Sigma-Aldrich). Forty million cells were injected using 3-ml 20-gauge syringes into four different injection sites (10 million per injection site) on the back of rhesus macaques, two below the scapulae and two above the iliac crest on either side of the spine. At each cell implantation site, the skin was pinched to create a tent, and cells were injected very slowly. Mild pressure was applied to the puncture wounds for approximately 10 s to prevent cell leakage. Buprenorphine SR (0.2 mg kg^−1^) and meloxicam SR (0.3 mg kg^−1^) were given subcutaneously for analgesia, and animals were returned to home housing.

#### Preparation of rhesus macaque wt^allo^ and HIP^allo^ iPSCs and injection into rhesus macaques

Two hundred million wt^allo^ and HIP^allo^ iPSCs were resuspended in 1.2 ml of RPMI 1640 media (Thermo Fisher Scientific) including pro-survival cocktail as described above. Cells were divided into five 1-ml syringes without dead volume with an 18-gauge needle with 240 μl each. A 2-cm skin incision was made over the middle anterior side of the quadricep muscle, and the cells were injected in a starburst pattern. The incisions were closed with 5-0 absorbable suture using inverted intradermal stitches, and a circle was drawn with a permanent pen to mark the injection area. Buprenorphine SR (0.2 mg kg^−1^) and meloxicam SR (0.3 mg kg^−1^) were given subcutaneously for analgesia, and animals were returned to home housing. All animals received methylprednisolone starting 1 d before the cell injection at 30 mg kg^−1^, which was then tapered to 10 mg kg^−1^, 6 mg kg^−1^, 3 mg kg^−1^ and 1.5 mg kg^−1^ for 3 d each.

#### Transplantation of rhesus macaque wt^allo^ and HIP^allo^ islet clusters into rhesus macaques

Fifteen million wt^allo^ or HIP^allo^ FLuc^+^ islet cells were transplanted as re-aggregated islet clusters into the quadricep muscle of rhesus macaques. Islet clusters were resuspended in 100 μl of RPMI 1640 media (Thermo Fisher Scientific) including pro-survival cocktail as described above and loaded into 1-ml syringes without dead volume with a 23-gauge needle. A 2-cm skin incision was made over the middle anterior side of the quadricep muscle, and the cells were injected in a string pattern parallel to the muscle fibers. The incisions were closed with 5-0 absorbable suture using inverted intradermal stitches, and a circle was drawn with a permanent pen to mark the injection area. Buprenorphine SR (0.2 mg kg^−1^) and meloxicam SR (0.3 mg kg^−1^) were given subcutaneously for analgesia, and animals were returned to home housing.

#### BLI

For BLI, d-luciferin firefly potassium salt (375 mg kg^−1^, Biosynth) dissolved in sterile PBS (pH 7.4, Gibco, Invitrogen) was injected intravenously into anesthetized rhesus macaques. Animals were imaged using the Lago (Spectral Instruments Imaging) 12 min after the d-luciferin application for an exposure of 5 min. Region of interest bioluminescence was quantified in units of maximum p/s/cm^2^/sr. The maximum signal from a region of interest was measured using Aura 3.2 software (Spectral Instruments Imaging).

#### ELISpot

For uni-directional ELISpot assays, recipient PBMCs were isolated from rhesus macaques at different timepoints, and CD3^+^ T cells were sorted (BD FACSAria Fusion). Donor cells were treated with mitomycin (50 μg ml^−1^ for 30 min, Sigma-Aldrich) and used as stimulator cells. In total, 1 × 10^5^ stimulator cells were incubated with 5 × 10^5^ recipient responder T cells for 36 h, and IFN-γ spot frequencies were enumerated using an ELISpot plate reader with ImmunoSpot 7.0 software (AID Diagnostika).

#### Total IgM and IgG ELISA

The total rhesus IgM ELISA kit (MyBioSource) and the total rhesus IgG ELISA kit (Molecular Innovations) were used to measure total IgM or IgG in monkey serum. Samples were diluted and pipetted according to the manufacturer’s instructions. In brief, standards and samples were added to pre-coated 96-well ELISA plates and incubated for 1 h. After the removal of unbound proteins by washing, anti-IgM or anti-IgG antibodies conjugated with HRP were added. These enzyme-labeled antibodies form complexes with the previously bound IgM or IgG. The enzyme bound to the immunosorbent is assayed by the addition of a chromogenic substrate, tetramethylbenzidine. Samples were analyzed in a microplate reader with Kaleido 3.0 software (Thermo Fisher Scientific).

#### DSA

Sera from recipient animals were de-complemented by heating to 56 °C for 30 min. Equal amounts of sera and cell suspensions (5 × 10^6^ per milliliter) were incubated for 45 min at 4 °C. Cells were labeled with FITC-conjugated goat anti-rhesus IgM (BioLegend) or FITC-conjugated goat anti-rhesus IgG (Southern Biotech), and MFI was analyzed by flow cytometry (Attune).

#### Rhesus macaque NK cell isolation

Rhesus PBMCs were sorted on the FACSAria Fusion using FITC-conjugated anti-CD8 (clone LT8, ab28010, 1:5 dilution, Abcam) and PE-conjugated anti-NKG2A (clone REA110, 130-114-092, 1:50 dilution, Miltenyi Biotec) antibodies to select a CD8^+^NKG2A^+^ NK cell population.

#### Macrophage differentiation from PBMCs

PBMCs were isolated by Ficoll separation from fresh blood and resuspended in RPMI 1640 with 10% heat-inactivated FCS hi and 1% penicillin–streptomycin (all Gibco). Cells were plated in 24-well plates at a concentration of 1 × 10^6^ cells per milliliter, and 10 ng ml^−1^ rhesus M-CSF (Biorbyt) was added. Media was changed every other day until day 6. Macrophages were stimulated from day 6 with 1 μg ml^−1^ rhesus IL-2 (MyBiosource) for 24 h before the cells were used.

#### PBMC, T cell, NK cell and macrophage killing assays on the xCELLigence platform

Rhesus macaque PBMC, T cell, NK cell and macrophage killing assays were performed on the xCELLigence MP platform (ACEA Biosciences). Specialized 96-well E-plates (ACEA Biosciences) were coated with collagen (Sigma-Aldrich), and 4 × 10^4^ target iPSCs were plated in 100 μl of media. After the cell index reached 0.7, the effector cells were added at an E:T ratio of 1:1. NK cells were stimulated with 1 μg ml^−1^ human IL-2 (PeproTech). As killing control, cells were treated with 2% Triton X-100 in water. Data were standardized and analyzed with RTCA 2.1 software (ACEA Biosciences).

#### CDC and ADCC killing by xCELLigence

CDC and ADCC killing assays were also performed on the xCELLigence MP platform. For CDC assays, 100 μl of untreated, complement-containing serum (1:1 mixed with media) was added. For ADCC assays, 50 μl of heat-inactivated serum with 4 × 10^4^ rhesus macaque NK cells or macrophages in 50 μl media were added. As killing control, cells were treated with 2% Triton X-100 in water. As survival control, cells were incubated only with media. Data were standardized and analyzed with RTCA 2.1 software (ACEA Biosciences).

#### Speed of killing in xCELLigence assays

The xCELLigence real-time impedance killing assay provides data about the kinetics of target cell killing, not only a binary yes or no readout. The speed of killing reflects the aggressiveness of the immune response and, thus, provides additional insight into the activation of this arm of the immune system and how vigorous the response is. The speed of target cell killing is expressed as the killing t_1/2_, the time until the normalized cell index drops from 1.0 to 0.5. Rapid killing took place within minutes; slow killing could take days. Because graphically the left *y* axis shows the % of target cell killing at the end of the assay (the higher the value, the more killing), we plotted killing t_1/2_^−1^ on the right axis (the higher the value, the faster the killing).

#### Statistics

All data are expressed as mean ± s.d. or mean ± s.e.m. Intergroup differences were appropriately assessed by either two-sided unpaired Student’s *t*-test or one-way analysis of variance (ANOVA) with Bonferroni post hoc test. Statistical analysis was performed on Prism 9 (GraphPad) or Excel 2019 (Microsoft).

### Reporting summary

Further information on research design is available in the Nature Portfolio Reporting Summary linked to this article.

## Online content

Any methods, additional references, Nature Portfolio reporting summaries, source data, extended data, supplementary information, acknowledgements, peer review information; details of author contributions and competing interests; and statements of data and code availability are available at 10.1038/s41587-023-01784-x.

### Supplementary information

Supplementary Information Supplementary Figs. 1–18.

Reporting Summary

## Data Availability

The authors declare that all data supporting the findings of this study are available within the paper and its supplementary information files. Supplementary information is available in the online version of the paper.
