# Supplementary material for: Hypoimmune induced pluripotent stem cells survive long term in fully immunocompetent, allogeneic rhesus macaques
Source: Nat Biotechnol. 2023 May 8;42(3):413–23. doi: 10.1038/s41587-023-01784-x (PMC10940156; doi:10.1038/s41587-023-01784-x)
Supplement: Supplementary file 1 — Supplementary Figs. 1–18. [file 41587_2023_1784_MOESM1_ESM.pdf]

nature biotechnology

Article https://doi.org/10.1038/s41587-023-01784-x

Hypoimmune induced pluripotent stem cells survive long term in fully immunocompetent, allogeneic rhesus macaques

In the format provided by the authors and unedited

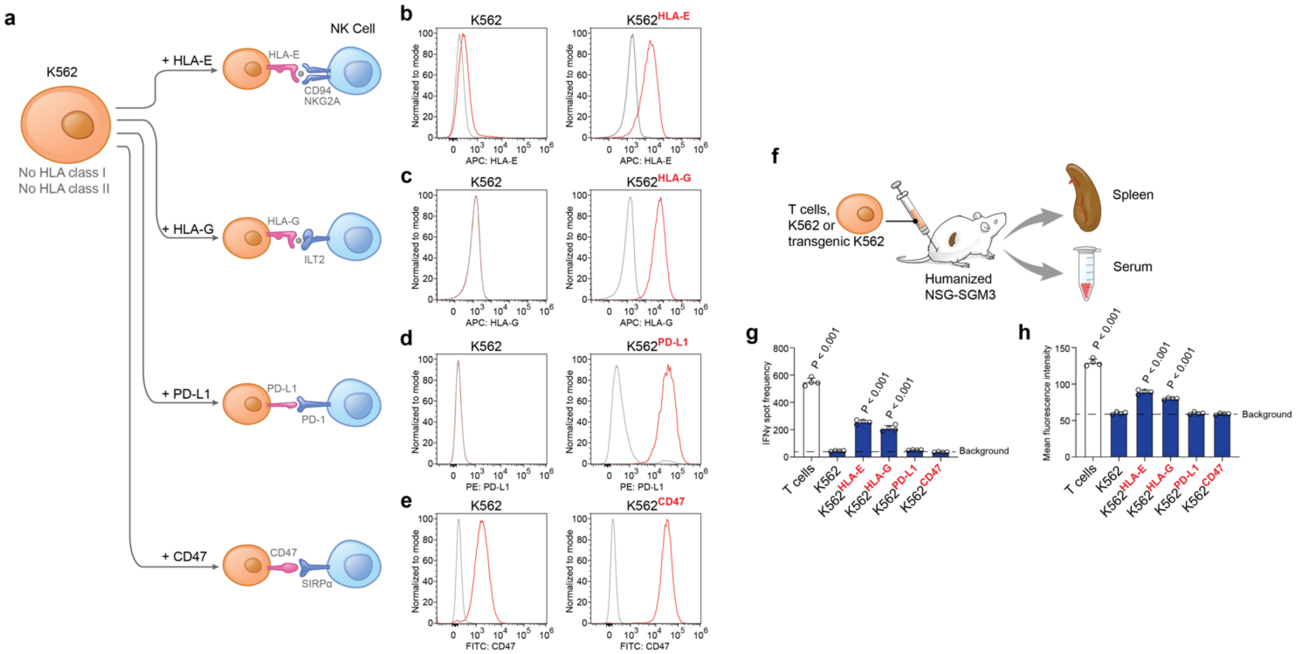

Supplementary Fig. 1: *HLA-E and HLA-G contribute to adaptive allo-immunity*. **a**, HLA class I and II-deficient K562 were transduced to express monomeric HLA-E or HLA-G or one of the immune-regulatory molecules PD-L1 or CD47, all of which have known inhibitory receptors on NK cells. **b-e**, Flow cytometry showed markedly increased surface expression of HLA-E (b), HLA-G (c), PD-L1 (d), or CD47 (e) on the transgenic K562 lines (representative histograms of 3 independent experiments). **f**, Human T cells, K562, or one of the transgenic K562 lines were intramuscularly injected into humanized NSG-SGM3 mice and after 6 days, splenocytes and serum were recovered. **g**, Splenocytes were re-challenged with the injected donor cells in Elispot assays and IFN-γ release was measured by spot frequency (mean ± s.d., 4 mice per group, one-way ANOVA with Bonferroni postHoc comparison to the K562 group). Responder cells from humanized mice that received no cell transplants are shown as background. **h**, The injected donor cells were incubated with serum and a FITC-labeled anti-IgM antibody. Donor cell-specific antibodies (DSA) of the IgM type were quantified by mean fluorescence intensity (mean ± s.d., 4 mice per group, one-way ANOVA with Bonferroni postHoc comparison to the K562 group). K562 incubated with serum from humanized mice that received no cell transplants are shown as background.

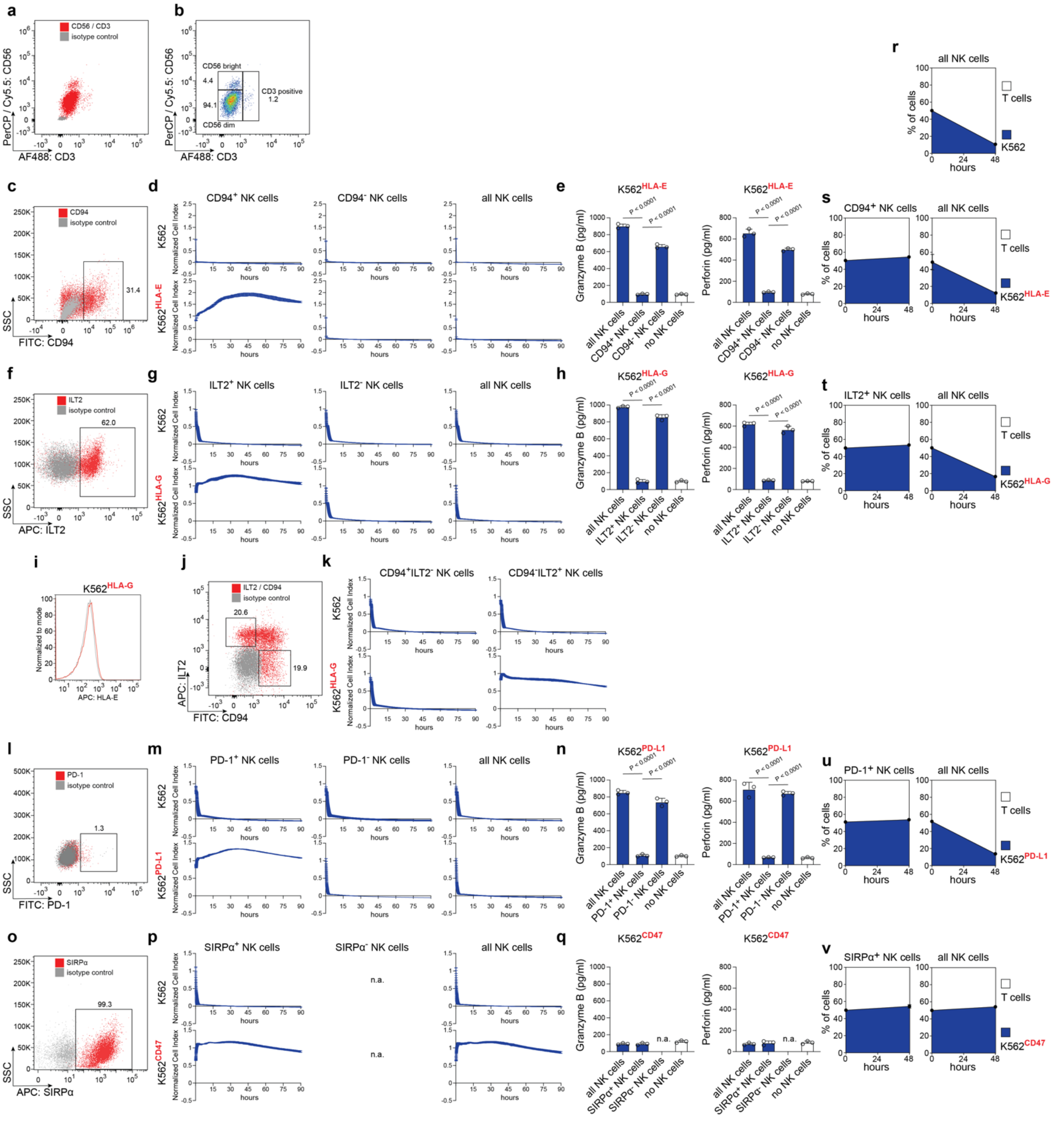

Supplementary Fig. 2: *CD47 provides broad protection for HLA-deficient K562 against killing by primary human NK cells*. **a**, Primary human NK cells isolated from PBMCs using negative immunomagnetic separation. **b**, NK cells were CD56^{+}, mainly CD56^{dim}, and the CD3^{+} contamination was low. **c**, IL-2 stimulated NK cells were assessed for their expression of the HLA-E receptor subunit CD94 and sorted into CD94^{+} and CD94^{-} populations. **d**, Killing assays of K562 and K562$^{HLA\text{-}E}$ with fractions of CD94^{+} or CD94^{-} NK cells and the whole unsorted population of human NK cells (mean ± s.d., triplicates for all time points). **e**, Granzyme B and perforin release assays of K562 and K562$^{HLA\text{-}E}$ with CD94^{+}, CD94^{-}, and all

NK cells (mean ± s.d., triplicates for all groups, one-way ANOVA with Bonferroni post-Hoc test). **f**, IL-2 stimulated NK cells were assessed for their expression of the HLA-G receptor ILT2 and sorted into ILT2^{+} and ILT2^{-} populations. **g**, Killing assays of K562 and K562^{HLA-G} with fractions of ILT2^{+} or ILT2^{-} NK cells and unsorted human NK cells (mean ± s.d., triplicates for all time points). **h**, Granzyme B and perforin release assays of K562 and K562^{HLA-G} with ILT2^{+}, ILT2^{-}, and all NK cells (mean ± s.d., triplicates for all groups, one-way ANOVA with Bonferroni post-Hoc test). **i**, Flow cytometry showed no increase in surface expression of HLA-E on K562^{HLA-G}. **j**, IL-2 stimulated NK cells were sorted into CD94^{+}ILT2^{-} and CD94^{-}ILT2^{+} populations. **k**, Killing assays of K562 and K562^{HLA-G} with fractions of CD94^{+}ILT2^{-} or CD94^{-}ILT2^{+} NK cells (mean ± s.d., triplicates for all time points). **l**, IL-2 stimulated NK cells were assessed for their expression of the PD-L1 receptor PD-1 and sorted into PD-1^{+} and PD1^{-} populations. **m**, Killing assays of K562 and K562^{PD-L1} with fractions of sorted PD-1^{+} or PD-1^{-} NK cells and unsorted human NK cells (mean ± s.d., triplicates for all time points). **n**, Granzyme B and perforin release assays of K562 and K562^{PD-L1} with PD-1^{+}, PD-1^{-}, and all NK cells (mean ± s.d., triplicates for all groups, one-way ANOVA with Bonferroni post-Hoc test). **o**, IL-2 stimulated NK cells were assessed for their expression of the CD47 receptor SIRPα. Since almost all NK cells were SIRPα^{+}, no SIRPα^{-} population could be enriched. **p**, Killing assays of K562 and K562^{CD47} with SIRPα^{+} and unsorted human NK cells (mean ± s.d., triplicates for all time points). **q**, Granzyme B and perforin release assays of K562 and K562^{CD47} with SIRPα^{+} and all NK cells (mean ± s.d., triplicates for all groups, one-way ANOVA with Bonferroni post-Hoc test). **r-v**, A 1:1 mixture of human T cells with K562 (r), K562^{HLA-E} (s), K562^{HLA-G} (t), K562^{PD-L1} (u), or K562^{CD47} (v) was injected into the peritoneum of NSG mice. The mice additionally received NK cells sorted to express the inhibitory receptor associated with the K562 transgene or they received unsorted human NK cells. After 48 hours, the proportion of remaining intraperitoneal graft cells was assessed (mean ± s.d., quadruplicates of 3 mice per group). All flow cytometry graphs are representatives of 2 independent experiments.

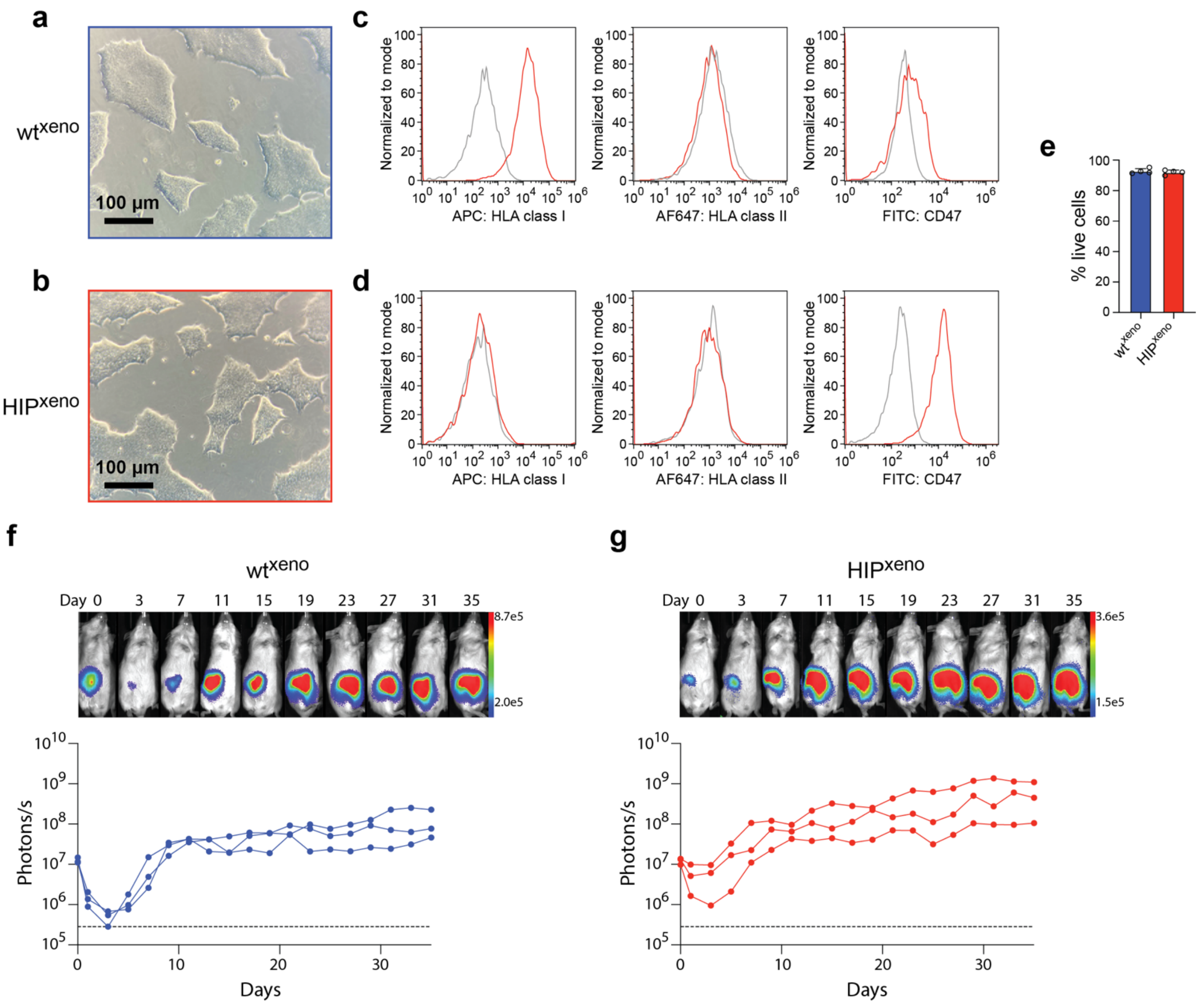

Supplementary Fig. 3: *Characterization of human wt and HIP cells before xenogeneic transplantation into rhesus macaques*. **a-b**, The morphology of wt^{xeno} (a) and HIP^{xeno} (b) cultures are shown (representative images of 3 independent experiments). **c-d**, Surface expression of HLA class I and class II and CD47 on wt^{xeno} (c) and HIP^{xeno} (d) was assessed by flow cytometry (representative histograms of 3 independent experiments). **e**, The viability of the cell preparations of wt^{xeno} and HIP^{xeno} before transplantation into rhesus macaques was above 90% (mean ± s.d., 4 independent experiments per group). **f-g**, One million wt^{xeno} (f) or HIP^{xeno} (g) were subcutaneously injected into NSG mice and their survival and proliferation was quantitatively assessed with BLI (3 mice per group, individual mice are shown).

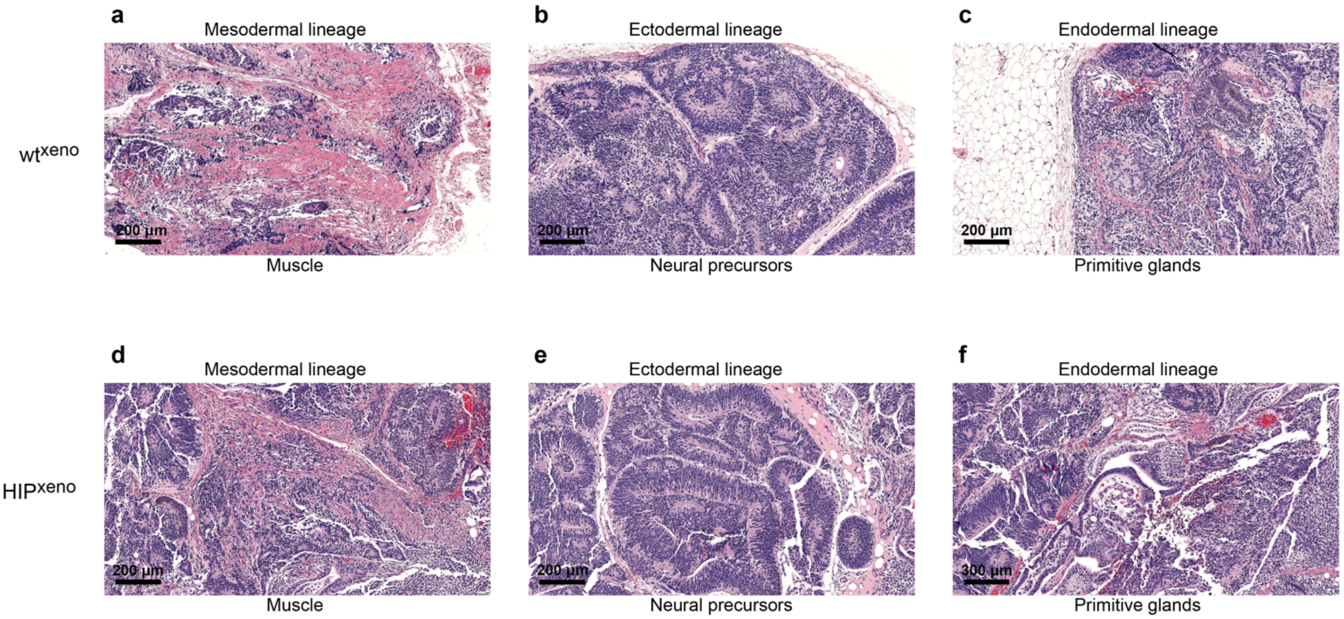

Supplementary Fig. 4: *Human wt and HIP iPSCs form tissues of all 3 germ layers.* **a-c**, Teratomas formed by wt^{xeno}. Representative pictures of mesodermal (a), ectodermal (b), and endodermal tissues (c) of at least two independent sections are shown. **d-f**, Teratomas formed by HIP^{xeno}. Representative pictures of mesodermal (d), ectodermal (e), and endodermal tissues (f) of at least two independent sections are shown.

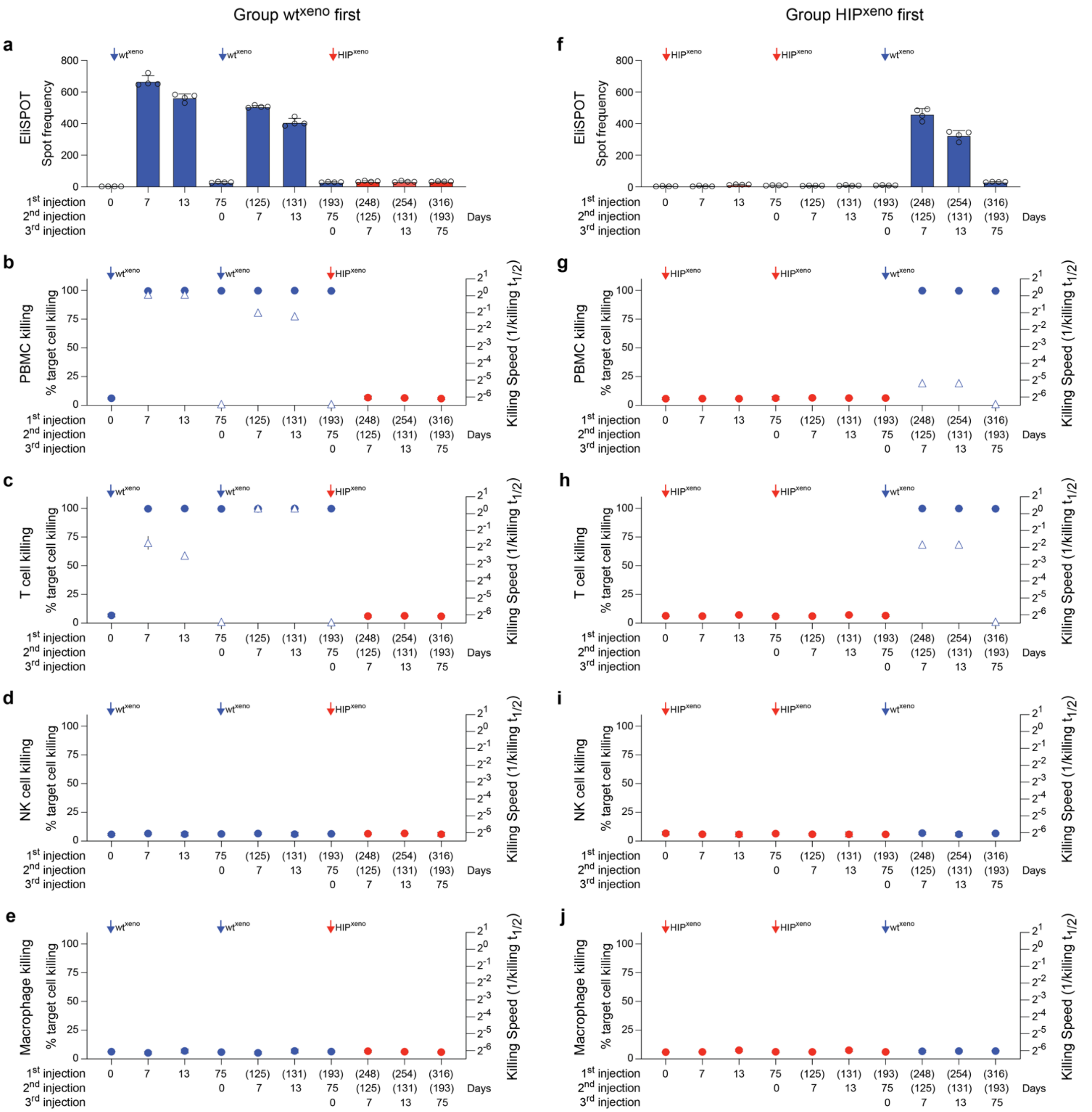

Supplementary Fig. 5: *Cellular immune responses against xenogeneic human wt and HIP grafts*. **a-e**, Immune assays in the group receiving wt^{xeno} first. Elispot assays with recipient monkey PBMCs drawn at scheduled time points (a, mean ± s.d., 4 monkeys). Killing assays with recipient monkey PBMCs (b), T cells (c), NK cells (d), and macrophages (e). Percent target cell killing is shown on the left y-axis (l, mean ± s.d.), killing speed on the right y-axis (r, killing $t_{1/2}^{-1}$, mean ± s.e.m., 4 monkeys). **f-j**, Immune assays in the group receiving HIP^{xeno} first. Elispot assays with recipient monkey PBMCs (f, mean ± s.d., 4 monkeys). Killing assays with recipient monkey PBMCs (g), T cells (h), NK cells (i), and macrophages (j). Percent target cell killing is shown on the left y-axis (l, mean ± s.d.), killing speed on the right y-axis (r, killing $t_{1/2}^{-1}$, mean ± s.e.m., 4 monkeys). All assays run against wt^{xeno} and HIP^{xeno} are shown in blue and red, respectively.

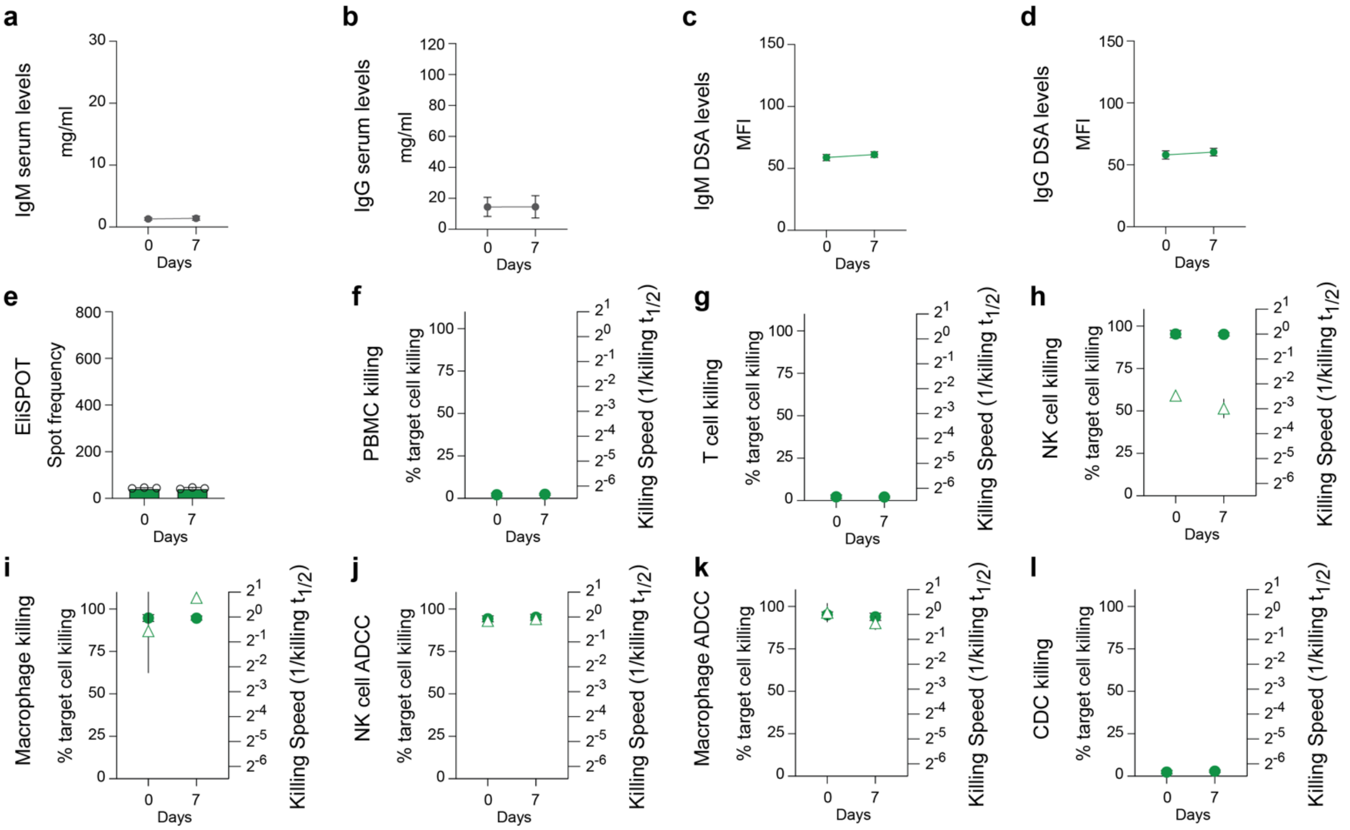

Supplementary Fig. 6: *Immune responses against xenogeneic human DKO grafts*. **a-d**, Total serum IgM (a) and IgG (b) levels, and DSA IgM (c) and IgG (d) levels are shown (mean ± s.d., 3 monkeys). **e**, Elispot assays with recipient monkey PBMCs drawn at scheduled time points (mean ± s.d., 3 monkeys). **f-i**, Killing assays with recipient monkey PBMCs (f), T cells (g), NK cells (h), and macrophages (i). Percent target cell killing is shown on the left y-axis (mean ± s.d.), killing speed on the right y-axis (killing $t_{1/2}^{-1}$, mean ± s.e.m., 3 monkeys). **j-l**, ADCC assays with de-complemented recipient monkey serum and NK cells (j) or macrophages (k) and CDC assays with complete recipient monkey serum (l) are shown. Percent target cell killing is shown on the left y-axis (mean ± s.d.), killing speed on the right y-axis (killing $t_{1/2}^{-1}$, mean ± s.e.m., 3 monkeys).

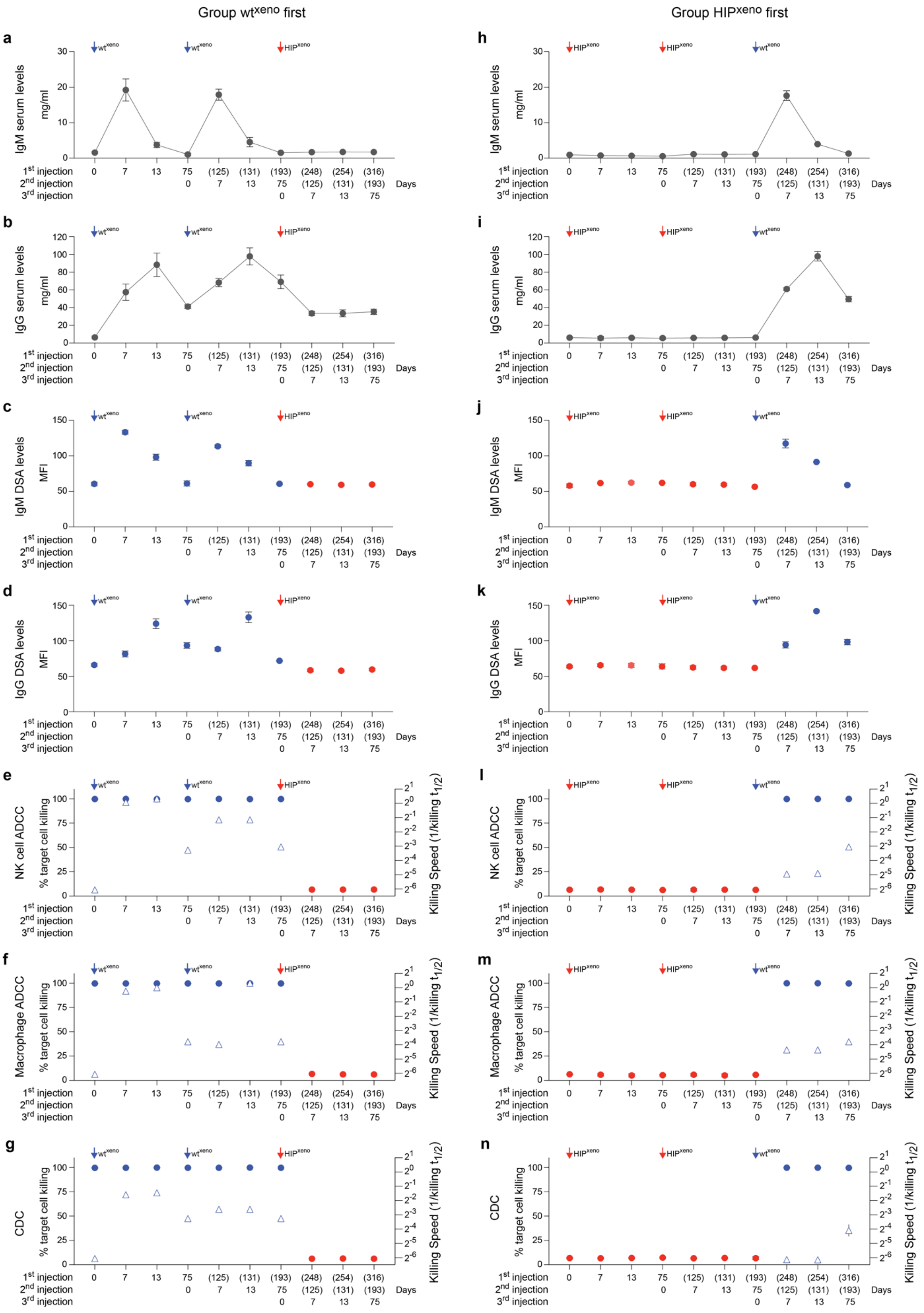

Group wtxeno first
Group HIPxeno first
a
b
c
d
e
f
g
h
i
j
k
l
m
n
wtxeno
HIPxeno
IgM serum levels
IgG serum levels
mg/ml
IgM DSA levels
IgG DSA levels
MFI
NK cell ADCC
Macrophage ADCC
CDC
% target cell killing
Killing Speed (1/killing t1/2)
1st injection 0 7 13 75 (125) (131) (193) (248) (254) (316)
2nd injection 0 7 13 75 (125) (131) (193) Days
3rd injection 0 7 13 75

Supplementary Fig. 7: *Antibody-mediated responses against xenogeneic human wt and HIP grafts*. **a-g**, Antibody immune assays in the group receiving wt^{xeno} first. Total serum IgM (a) and IgG (b) levels, and DSA IgM (c) and IgG (d) levels are shown (mean ± s.d., 4 monkeys). ADCC assays with de-complemented recipient monkey serum and NK cells (e) or macrophages (f) and CDC assays with complete recipient monkey serum (g) are shown. Percent target cell killing is shown on the left y-axis (l, mean ± s.d.), killing speed on the right y-axis (r, killing $t_{1/2}^{-1}$, mean ± s.e.m., 4 monkeys). **h-n**, Antibody immune assays in the group receiving HIP^{xeno} first. Total serum IgM (h) and IgG (i) levels, and DSA IgM (j) and IgG (k) levels are shown (mean ± s.d., 4 monkeys). ADCC assays with de-complemented recipient monkey serum and NK cells (l) or macrophages (m) and CDC assays with complete recipient monkey serum (n) are shown. Percent target cell killing is shown on the left y-axis (l, mean ± s.d.), killing speed on the right y-axis (r, killing $t_{1/2}^{-1}$, mean ± s.e.m., 4 monkeys). All assays run against wt^{xeno} and HIP^{xeno} are shown in blue and red, respectively.

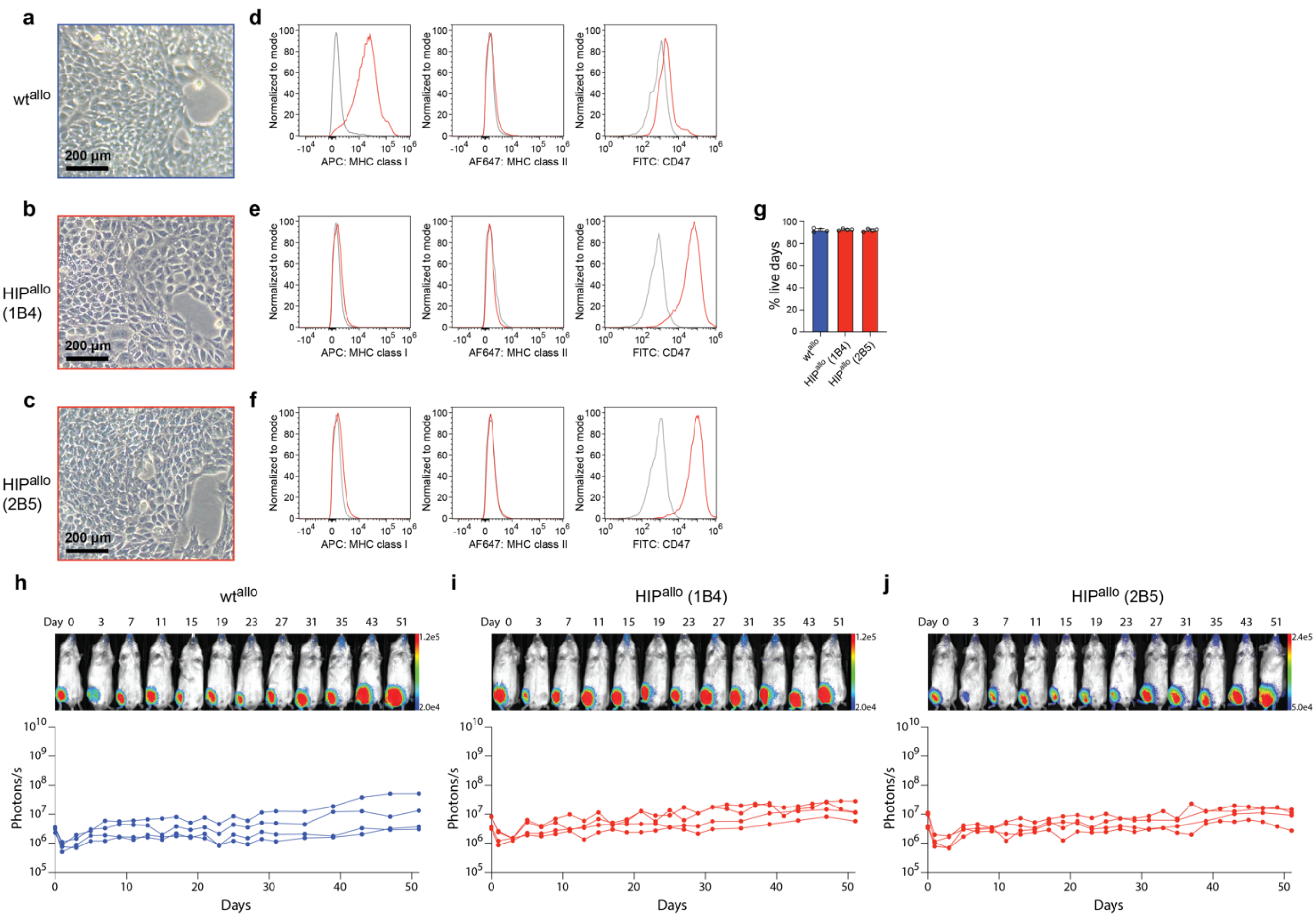

Supplementary Fig. 8: *Characterization of rhesus macaque wt and HIP iPSCs before allogeneic transplantation*. **a-c**, The morphology of wt^{allo} (a) and the two HIP^{allo} clones 1B4 (b) and 2B5 (c) are shown (representative images of 3 independent experiments). **d-f**, Surface expression of HLA class I and class II and CD47 on wt^{allo} (d), HIP^{allo} clone 1B4 (e) and HIP^{allo} clone 2B5 (f) as assessed by flow cytometry (representative histograms of 3 independent experiments). **g**, The viability of the cell preparations of wt^{xeno}, HIP^{allo} clone 1B4, and HIP^{allo} clone 2B5 before transplantation into rhesus macaques was above 90% (mean ± s.d., 4 independent experiments per group). **h-j**, Ten million wt^{allo} (h), HIP^{allo} clone 1B4 (i) and HIP^{allo} clone 2B5 (j) were subcutaneously injected into NSG mice and their survival and proliferation was quantitatively assessed with BLI (4 mice per group, individual mice are shown).

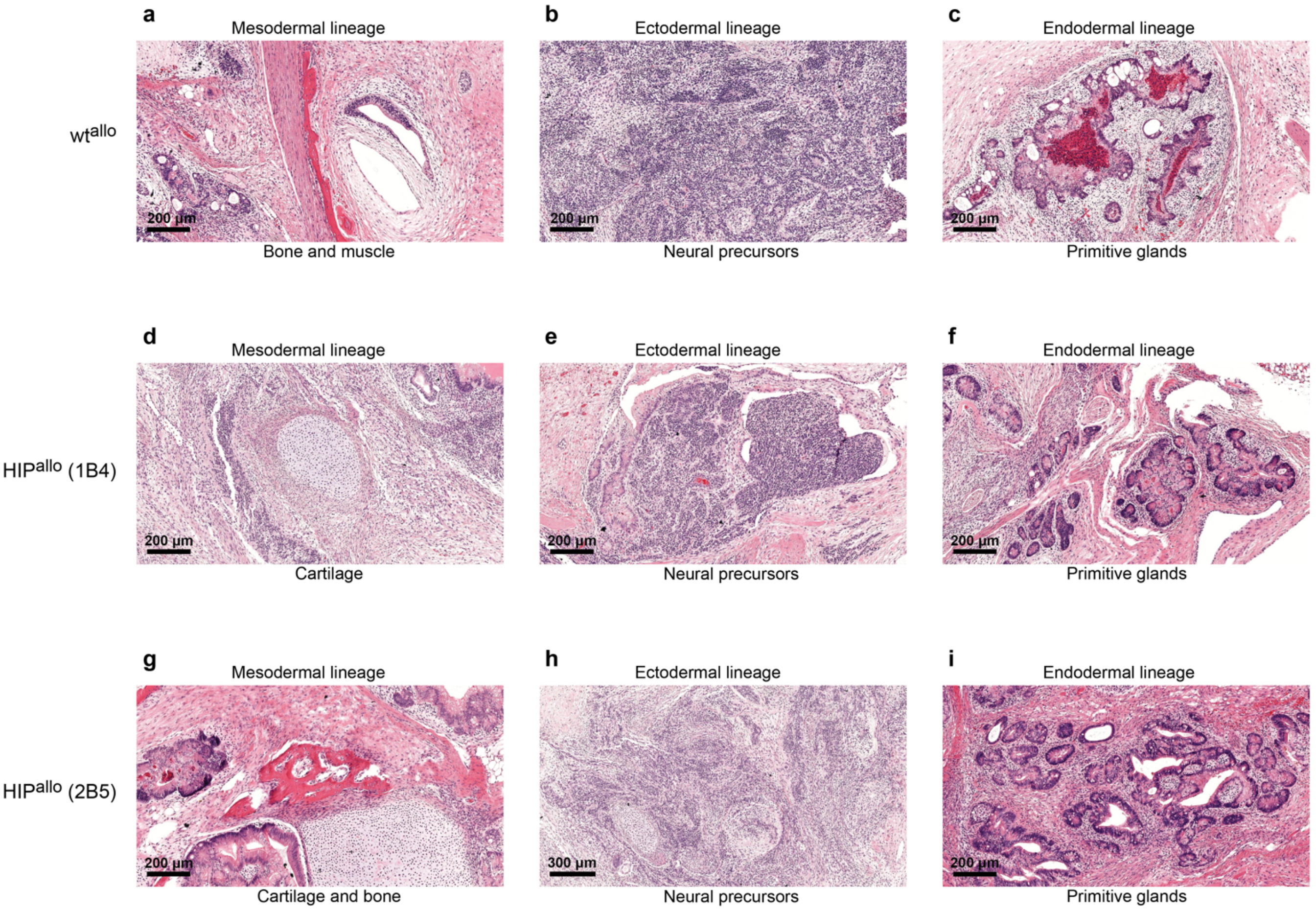

Supplementary Fig. 9: *Rhesus macaque wt and HIP iPSCs form tissues of all 3 germ layers.* **a-c**, Teratomas formed by wt^{allo}. Representative pictures of mesodermal (a), ectodermal (b), and endodermal tissues (c) of at least two independent sections are shown. **d-f**, Teratomas formed by HIP^{allo} clone 1B4. Representative pictures of mesodermal (d), ectodermal (e), and endodermal tissues (f) of at least two independent sections are shown. **g-i**, Teratomas formed by HIP^{allo} clone 2B5. Representative pictures of mesodermal (g), ectodermal (h), and endodermal tissues (i) of at least two independent sections are shown.

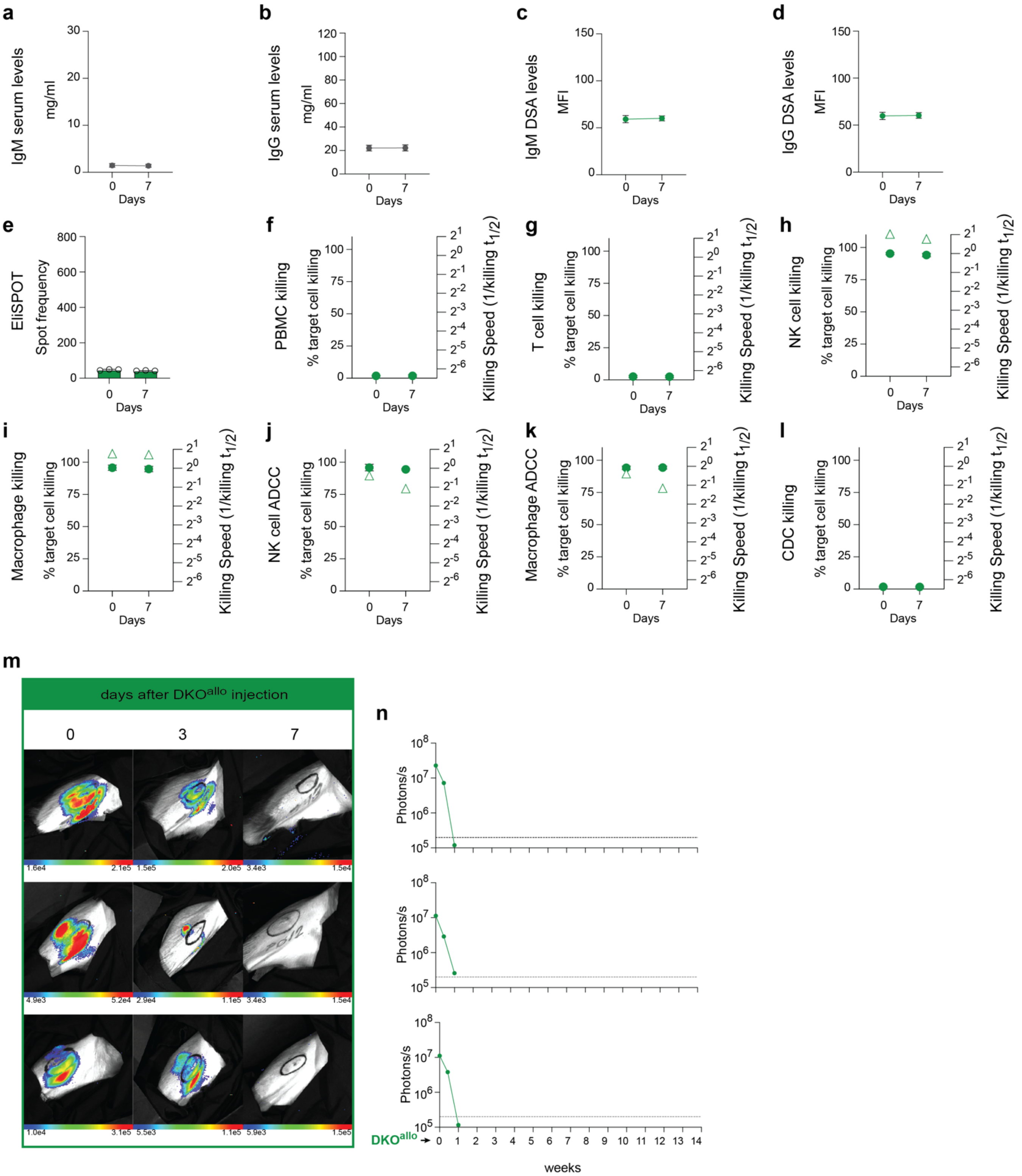

Supplementary Fig. 10: *Immune responses against allogeneic rhesus macaque DKO grafts*. **a-d**, Total serum IgM (a) and IgG (b) levels, and DSA IgM (c) and IgG (d) levels are shown (mean ± s.d., 3 monkeys). **e**, Elispot assays with recipient monkey PBMCs drawn at scheduled time points (mean ± s.d., 3 monkeys). **f-i**, Killing assays with recipient monkey PBMCs (f), T cells (g), NK cells (h), and macrophages

(i). Percent target cell killing is shown on the left y-axis (mean ± s.d.), killing speed on the right y-axis (killing $t_{1/2}^{-1}$, mean ± s.e.m., 3 monkeys). **j-l**, ADCC assays with de-complemented recipient monkey serum and NK cells (j) or macrophages (k) and CDC assays with complete recipient monkey serum (l) are shown. Percent target cell killing is shown on the left y-axis (mean ± s.d.), killing speed on the right y-axis (killing $t_{1/2}^{-1}$, mean ± s.e.m., 3 monkeys). **m-n**, BLI images and BLI signals over time are shown for all three rhesus macaques receiving DKO^{allo}.

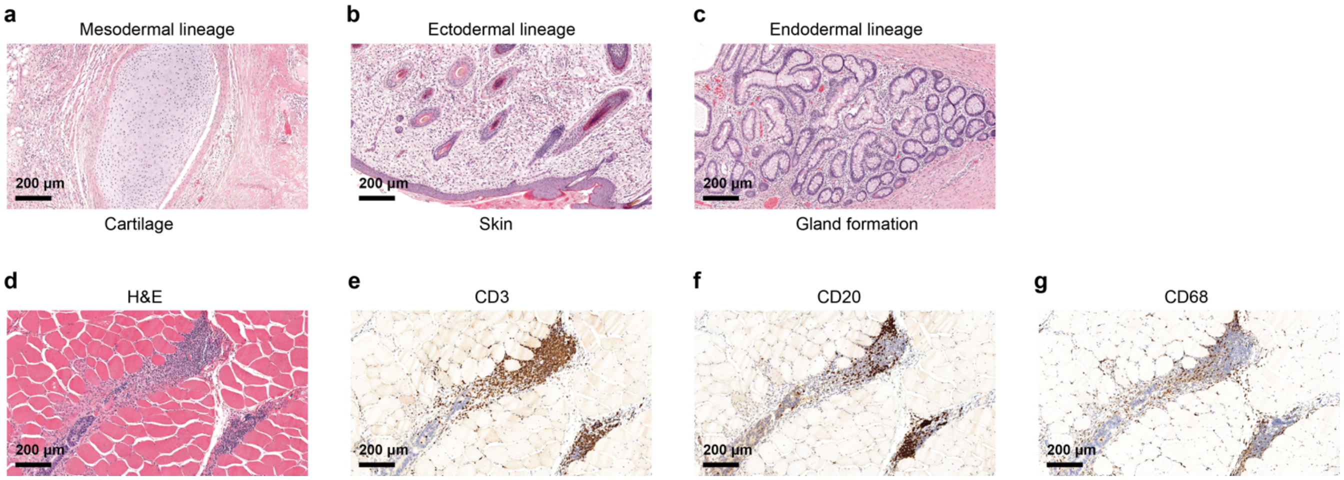

Supplementary Fig. 11: *Histology of intramuscular autologous rhesus macaque grafts.* **a-c**, Autologous iPSC injection site after 18 weeks at 100× magnification, which revealed well-differentiated teratomas with mature mesoderm (a), ectoderm (b), and endoderm structures (c). Representative images of at least two independent sections are shown. **d-g**, Representative images are shown from hematoxylin and eosin (H&E, d) staining and immunohistochemical staining for CD3 (T lymphocytes, e), CD20 (B lymphocytes, f), CD68 (macrophages, g). Teratomatous masses revealed no zones of necrosis, but some inflammation was present. Few macrophages were present, and the perivascular T and B lymphocytes had organized into lymphoid structures with follicular and peri-follicular zones. Representative images of at least two independent sections are shown.

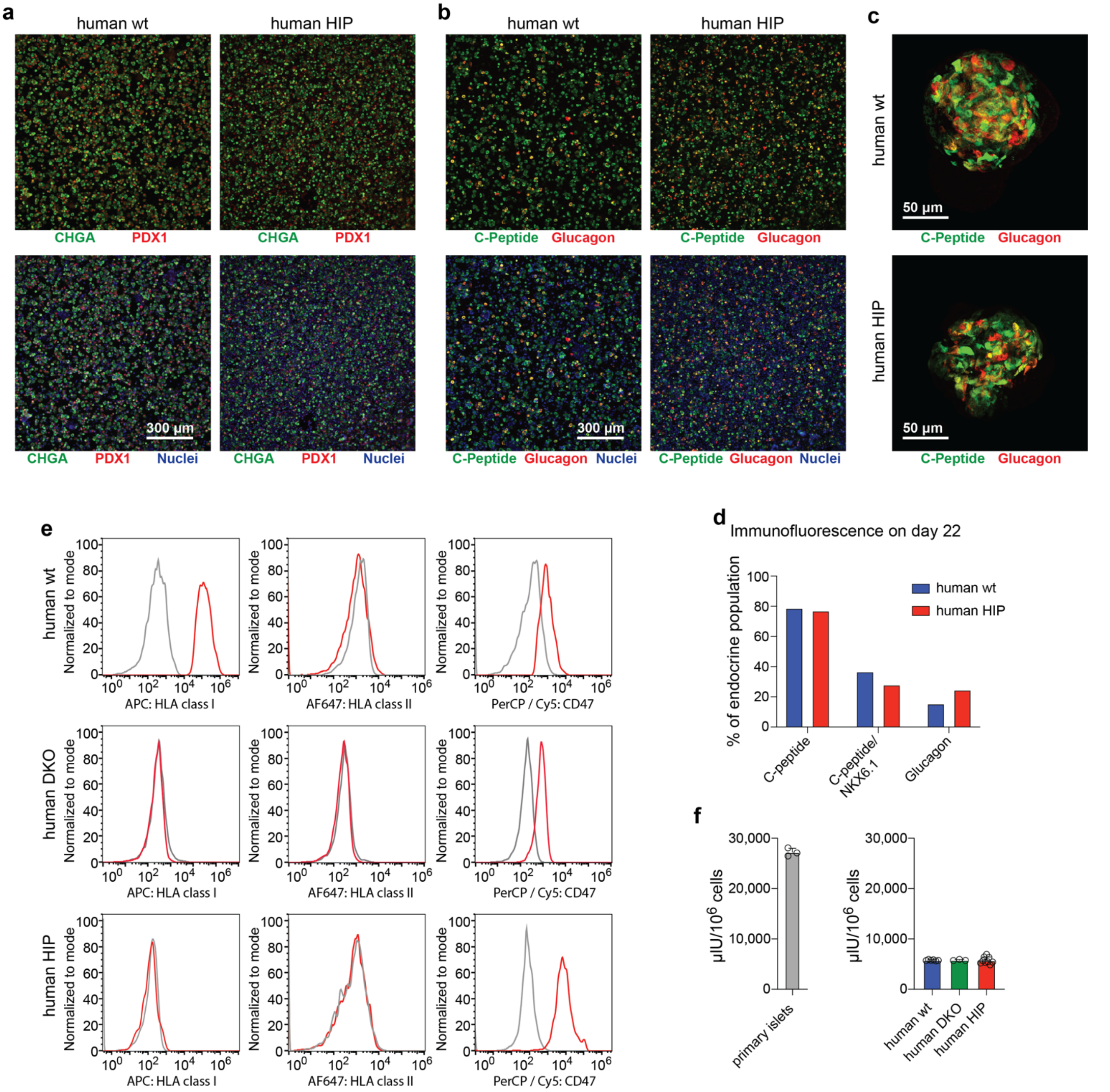

Supplementary Fig. 12: *Human HIP iPSCs can successfully be differentiated into human pancreatic islet cells.* **a**, Representative Immunofluorescence of CHGA (chromogranin A) and PDX1 (pancreatic and duodenal homeobox 1). CHGA marks all endocrine cells, and PDX1/CHGA marks all β and δ cells (and some polyhormonal cells). PDX1 cells without CHGA are pancreatic progenitors. Representative images of at least two independent experiments are shown. **b**, Representative Immunofluorescence of C-peptide and glucagon. The culture contained mostly cells expressing C-peptide, but there was a minority populations of glucagon^{+} cells. Representative images of at least two independent experiments are shown. **c**, Representative confocal images of whole islet cell clusters (representative pictures of two independent experiments). **d**, Quantitative assessment of differentiated islet cells from human wt and HIP (one experiment). **e**, Flow cytometry histograms for HLA class I and II and CD47 expression of wt,

DKO, and HIP islet cells (representative pictures of two independent experiments). **f**, Insulin release by one million primary islet cells (3 replicates) and wt (6 replicates), DKO (3 replicates), and HIP islet cells (9 replicates) (mean ± s.d.).

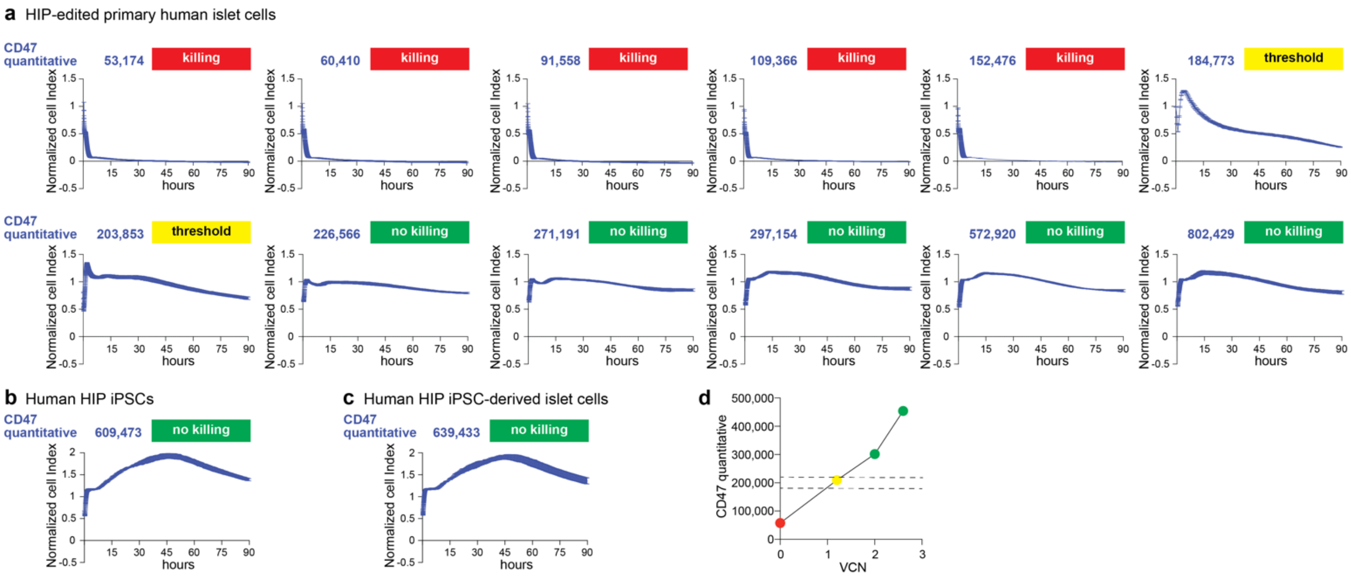

Supplementary Fig. 13: *CD47 threshold for human islet cells.* **a**, In twelve HIP-edited human primary islet cell cultures, CD47 expression levels were quantified using quantitative flow cytometry (one measurement per culture is shown). Impedance killing assays with human NK cells were subsequently performed (mean ± s.d., three independent replicates per group and time point). **b-c**, CD47 was quantified (one measurement per cell type) in human HIP iPSCs (b) and HIP iPSC-derived islet cells (c) and Impedance killing assays with human NK cells were performed (mean ± s.d., three independent replicates per group and time point). **d**, Unedited and HIP-edited islet cells were analyzed for quantitative CD47 expression and associated virus copy numbers (VCN). The colors of the dots were chosen according to the ranges defined in **a**.

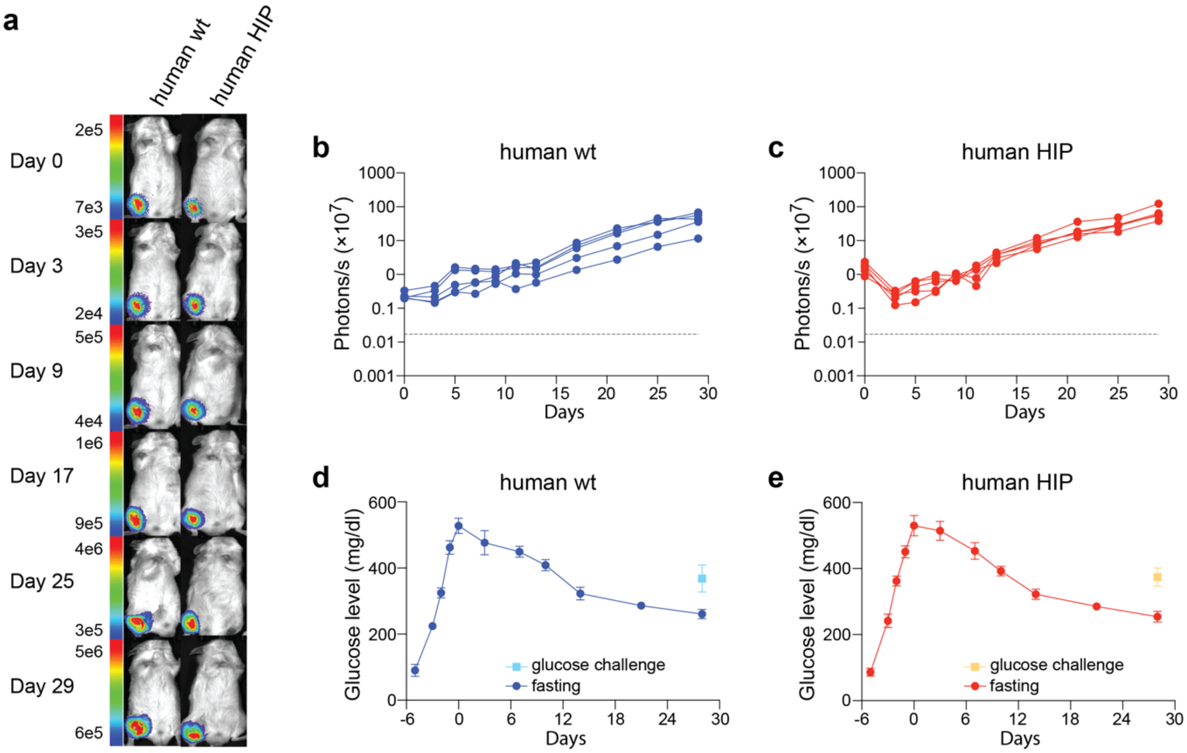

Supplementary Fig. 14: *Human wt and HIP islets similarly ameliorate diabetes in NSG mice.* **a-c**, NSG mice received streptozotocin and developed diabetes (a, one representative mouse is shown per group). Six days later, one thousand FLuc+ allogeneic wt (b, 5 animals), or HIP islet clusters (c, 8 animals) were injected intramuscularly and their survival was monitored with BLI (all individual animals are shown). **d-e**, Serial fasting blood glucose levels were quantified (mean ± s.d.) in the wt (d, 5 animals), or HIP group (e, 5 animals). On day 30, animals underwent a glucose challenge with blood draw 30 min later (mean ± s.d.).

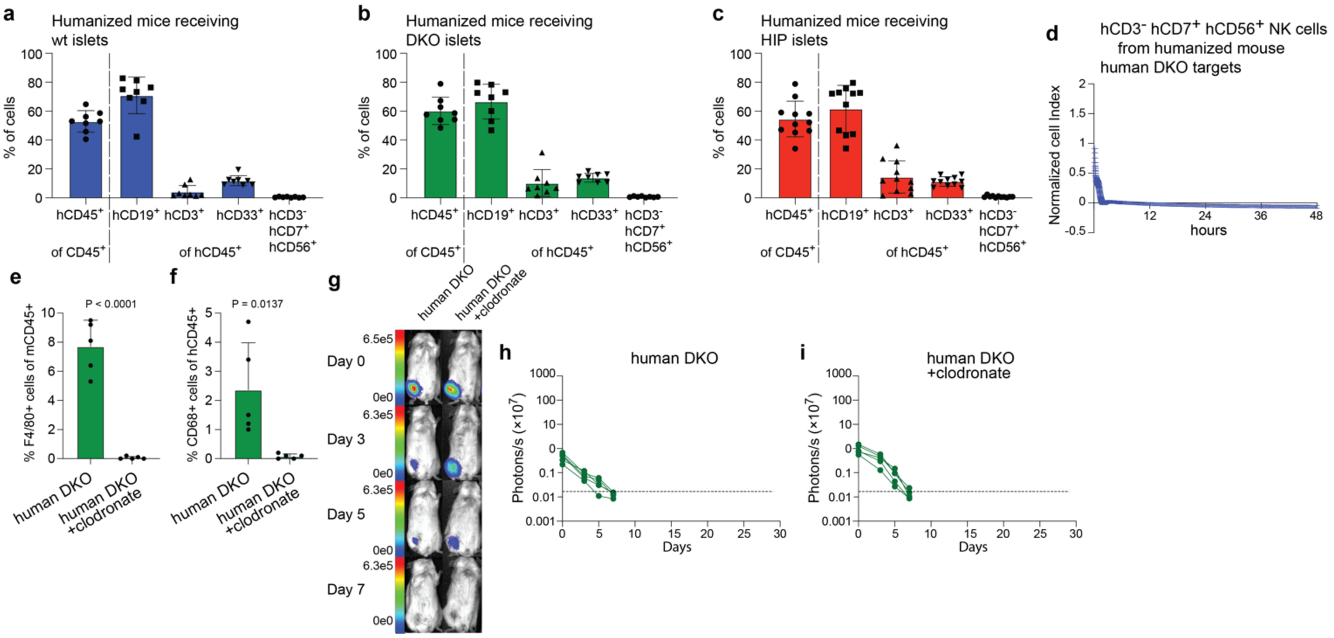

Supplementary Fig. 15: *Vanishing of human DKO grafts in allogeneic humanized mice*. **a-c**, Engraftment of human immune cells in humanized NSG-SGM3 mice before receiving wt (a, 8 animals), DKO (b, 8 animals), or HIP islet grafts (c, 11 animals) (mean ± s.d.). **d**, Impedance killing assay with human CD3$^-$ CD7$^+$CD56$^+$ NK cells isolated from the spleen of a humanized NSG-SGM3 mouse and human DKO iPSCs (mean ± s.d., three independent replicates per time point). **e-f**, Prevalence of human macrophages (e) and mouse macrophages (f) in humanized mice that received human DKO iPSC grafts without or with clodronate pre-treatment (4 animals in each group, mean ± s.d., two-sided *t*-test). **g-i**, BLI images (g) and BLI signals over time of humanized mice that received human DKO iPSC grafts without (h) or with clodronate pre-treatment (i, 4 animals in each group, all individual animals are shown).

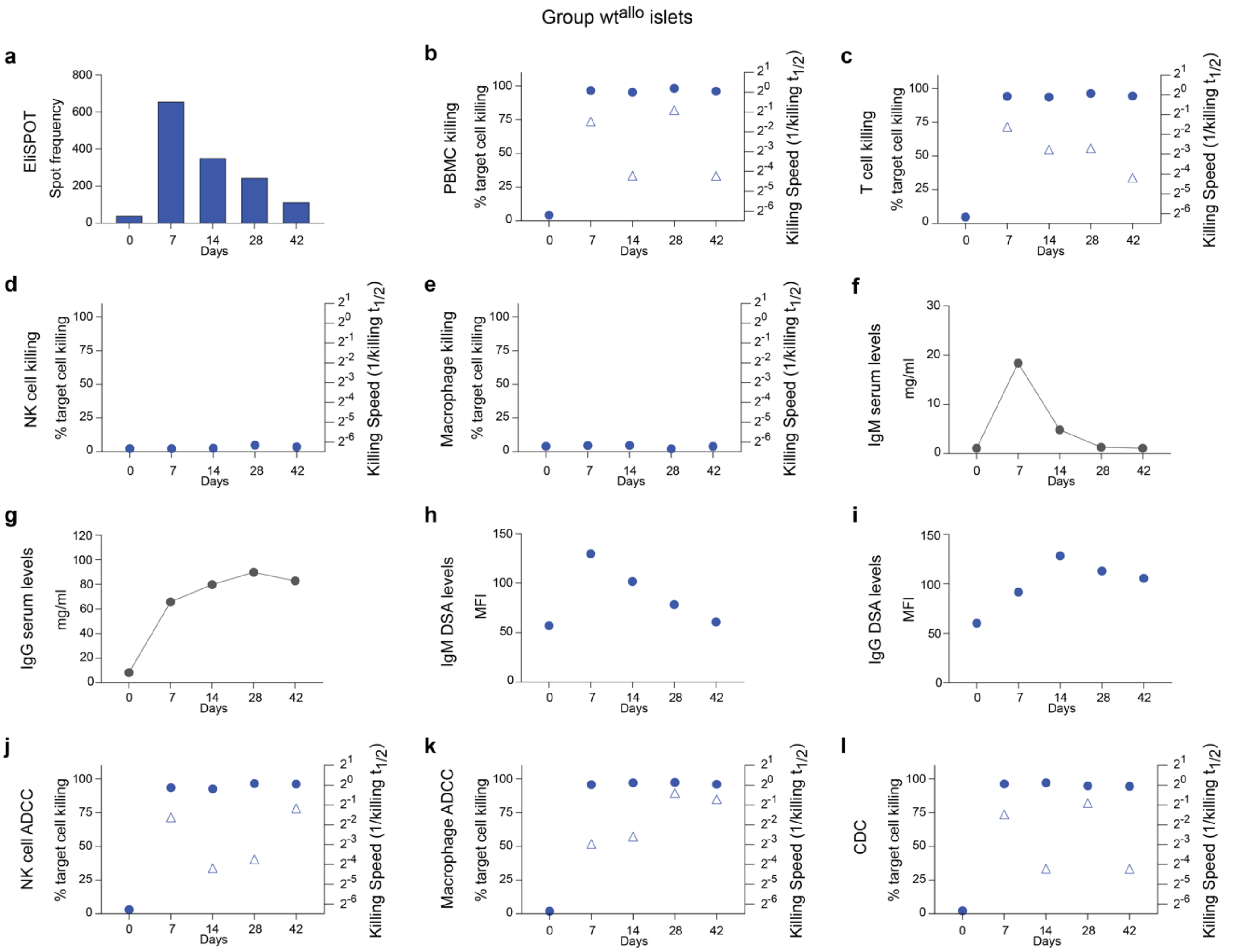

Supplementary Fig. 16: *Immune responses against allogeneic wt^{allo} islets*. **a**, Elispot assays with recipient monkey PBMCs drawn at scheduled time points. **b-e**, Killing assays with recipient monkey PBMCs (b), T cells (c), NK cells (d), and macrophages (e). Percent target cell killing is shown on the left y-axis, killing speed on the right y-axis (killing $t_{1/2}^{-1}$). **f-i**, Total serum IgM (f) and IgG (g) levels, and DSA IgM (h) and IgG (i) levels are shown. **j-l**, ADCC assays with de-complemented recipient monkey serum and NK cells (j) or macrophages (k) and CDC assays with complete recipient monkey serum (l) are shown. Percent target cell killing is shown on the left y-axis, killing speed on the right y-axis (killing $t_{1/2}^{-1}$). Data represent one monkey.

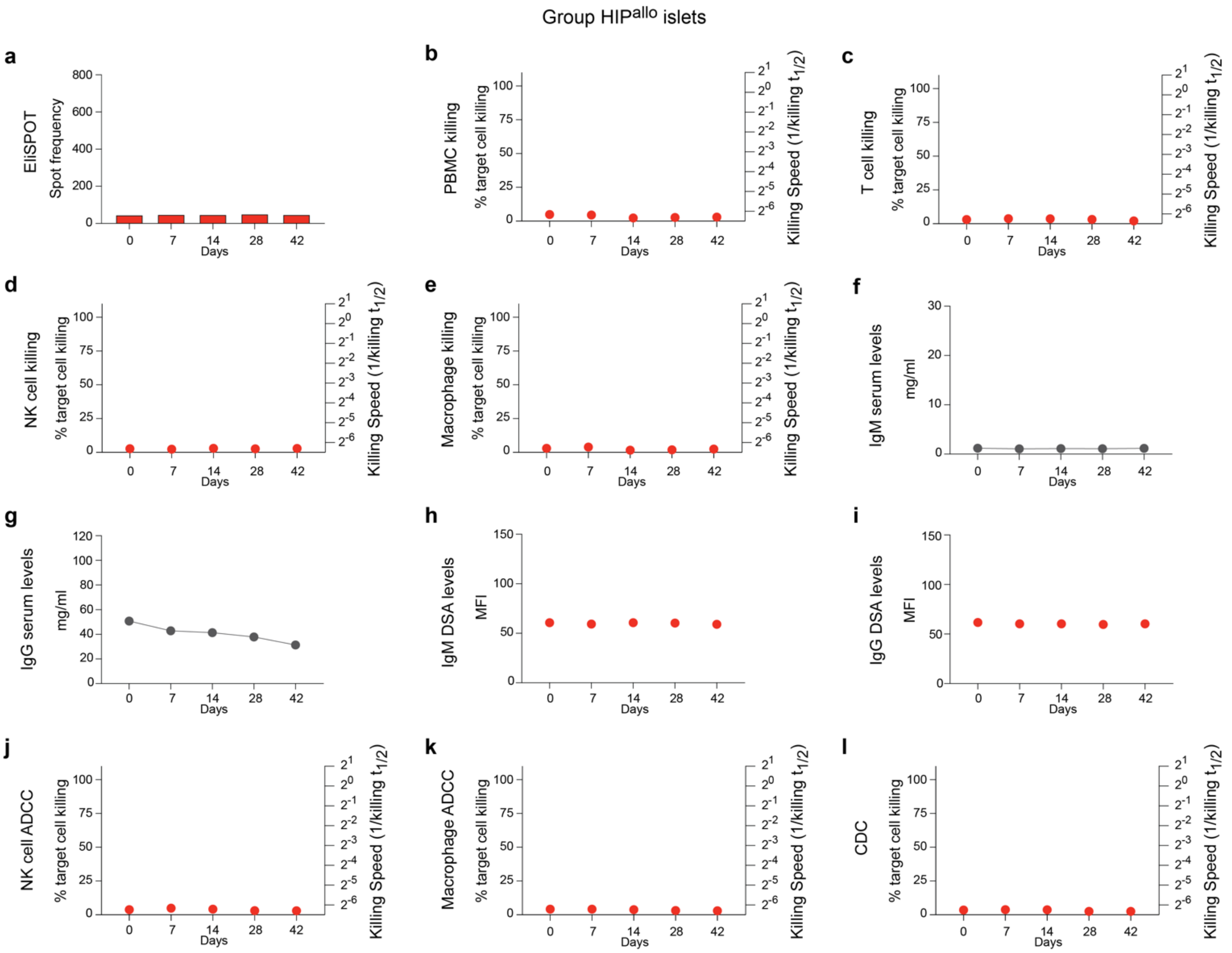

Supplementary Fig. 17: *Immune responses against allogeneic HIP^{allo} islets*. **a**, Elispot assays with recipient monkey PBMCs drawn at scheduled time points. **b-e**, Killing assays with recipient monkey PBMCs (b), T cells (c), NK cells (d), and macrophages (e). Percent target cell killing is shown on the left y-axis, killing speed on the right y-axis (killing $t_{1/2}^{-1}$). **f-i**, Total serum IgM (f) and IgG (g) levels, and DSA IgM (h) and IgG (i) levels are shown. **j-l**, ADCC assays with de-complemented recipient monkey serum and NK cells (j) or macrophages (k) and CDC assays with complete recipient monkey serum (l) are shown. Percent target cell killing is shown on the left y-axis, killing speed on the right y-axis (killing $t_{1/2}^{-1}$). Data represent one monkey.

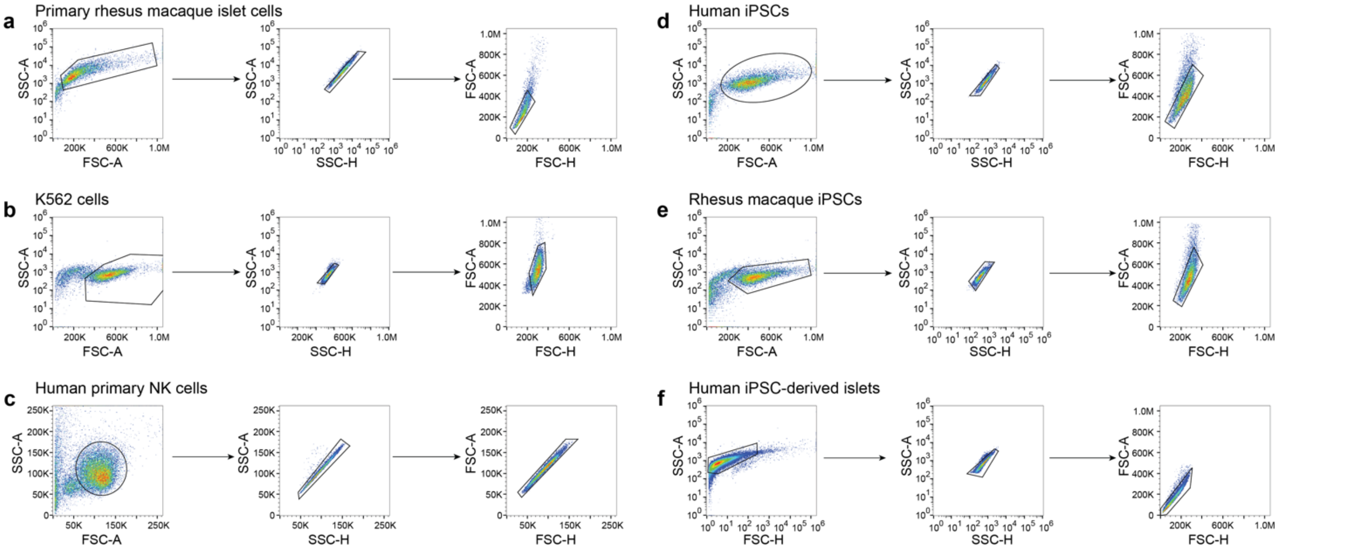

Supplementary Fig. 18: *Flow cytometry gating strategies*. **a**, One example showing the gating strategy for primary rhesus macaque islet cells (Fig. 6). **b**, One example showing the gating strategy for K562 cells (Supplementary Fig. 1b-e). **c**, One example showing the gating strategy for human primary NK cells (Supplementary Fig. 2a-c, f, i, j, l, o). **d**, One example showing the gating strategy for human iPSCs (Supplementary Fig. 3c-d). **e**, One example showing the gating strategy for rhesus macaque iPSCs (Supplementary Fig. 8d-f). **f**, One example showing the gating strategy for human iPSC-derived islets (Supplementary Fig. 12e).
