# Supplementary material for: Hypoimmune induced pluripotent stem cells survive long term in fully immunocompetent, allogeneic rhesus macaques
Source: Nat Biotechnol. 2023 May 8;42(3):413–23. doi: 10.1038/s41587-023-01784-x (PMC10940156; doi:10.1038/s41587-023-01784-x)
Supplement: Supplementary file 2 — Reporting Summary [file 41587_2023_1784_MOESM2_ESM.pdf]

nature portfolio

Corresponding author(s): Sonja Schrepfer

Last updated by author(s): Apr 24, 2023

Reporting Summary

Nature Portfolio wishes to improve the reproducibility of the work that we publish. This form provides structure for consistency and transparency in reporting. For further information on Nature Portfolio policies, see our Editorial Policies and the Editorial Policy Checklist.

Statistics

For all statistical analyses, confirm that the following items are present in the figure legend, table legend, main text, or Methods section.

n/a	Confirmed	
☐	☒	The exact sample size (*n*) for each experimental group/condition, given as a discrete number and unit of measurement
☐	☒	A statement on whether measurements were taken from distinct samples or whether the same sample was measured repeatedly
☐	☒	The statistical test(s) used AND whether they are one- or two-sided<br>*Only common tests should be described solely by name; describe more complex techniques in the Methods section.*
☒	☐	A description of all covariates tested
☒	☐	A description of any assumptions or corrections, such as tests of normality and adjustment for multiple comparisons
☐	☒	A full description of the statistical parameters including central tendency (e.g. means) or other basic estimates (e.g. regression coefficient) AND variation (e.g. standard deviation) or associated estimates of uncertainty (e.g. confidence intervals)
☒	☐	For null hypothesis testing, the test statistic (e.g. *F*, *t*, *r*) with confidence intervals, effect sizes, degrees of freedom and *P* value noted<br>*Give P values as exact values whenever suitable.*
☒	☐	For Bayesian analysis, information on the choice of priors and Markov chain Monte Carlo settings
☒	☐	For hierarchical and complex designs, identification of the appropriate level for tests and full reporting of outcomes
☒	☐	Estimates of effect sizes (e.g. Cohen's *d*, Pearson's *r*), indicating how they were calculated

Our web collection on statistics for biologists contains articles on many of the points above.

Software and code

Policy information about availability of computer code

Data collection	Attune NTx or BD FACSDiva 8.0.3 were used of the data collection of flow cytometry data. Aura 3.2 was used for quantification of bioluminescence imaging. Elispots were enumerated by Immunospot 7.0 software. XCelligence assays were measured with the RTCA 2.1 software. ELISA was measured with the Kaleido 3.0 software. Glucose data was measured on the Accu-Check Guide. Bio-Rad's QX Manager Software (Standard Edition, version 1.2) was used to estimate DelU3 and ARX reference genes
Data analysis	Data was automatically analyzed in the mentioned software above for Aura 3.2, RTCA 2.1, Kaleido 3.0 and Immunospot 7.0. Statistical analysis was performed on Prism 9. FlowJo 10 was used to analyze flow cytometric data. Prism 9 or Excel 2019 was used for graphing and statistical analysis.

For manuscripts utilizing custom algorithms or software that are central to the research but not yet described in published literature, software must be made available to editors and reviewers. We strongly encourage code deposition in a community repository (e.g. GitHub). See the Nature Portfolio guidelines for submitting code & software for further information.

Data

Policy information about availability of data

All manuscripts must include a data availability statement. This statement should provide the following information, where applicable:

- Accession codes, unique identifiers, or web links for publicly available datasets
- A description of any restrictions on data availability
- For clinical datasets or third party data, please ensure that the statement adheres to our policy

All data generated or analysed during this study are included in this published article (and its supplementary information files). No pre-established data exclusion method was used. No clinical data was included.

Field-specific reporting

Please select the one below that is the best fit for your research. If you are not sure, read the appropriate sections before making your selection.

☒ Life sciences ☐ Behavioural & social sciences ☐ Ecological, evolutionary & environmental sciences

For a reference copy of the document with all sections, see nature.com/documents/nr-reporting-summary-flat.pdf

Life sciences study design

All studies must disclose on these points even when the disclosure is negative.

Sample size	The sample size for the in vivo studies to achieve statistical significance was not calculated before the studies as the survival of the HIP cells in the different models was unknown prior. It was reasoned that 3-10 mice or 1-4 rhesus macaques per group in individual experiments would indicate valid efficacy. This was based on previous studies using HIP cells cells (Deuse T. Nat Biotechnol. 2019; 37:252-258). Sample sizes in vitro were determined by three or more samples for comparisons between one or multiple groups, followed by the statistical test. Again, the sample size to achieve statistical significance was not calculated before the studies for the reason described above.
Data exclusions	No pre-established data exclusion method was used.
Replication	Only assays that passed quality control were considered and were all successfully replicated.
Randomization	All samples were number coded until the readout was finalized. The numbers were assigned prior to the experiment and determined the group/ treatment/ condition. Animals were number coded and assigned to a group prior to the surgical procedure on an alternating basis.
Blinding	Group allocation for cell transplantations were performed by blinded investigators. For in vivo imaging and teratoma measurement, the investigators doing the readouts were not blinded, but not familiar with the experimental setup of this study and were not involved in data interpretation. For histology, the animal group that each cell type belonged to was unknown at the time to the individual doing the imaging.

Reporting for specific materials, systems and methods

We require information from authors about some types of materials, experimental systems and methods used in many studies. Here, indicate whether each material, system or method listed is relevant to your study. If you are not sure if a list item applies to your research, read the appropriate section before selecting a response.

Materials & experimental systems

n/a	Involved in the study	
☐	☒	Antibodies
☐	☒	Eukaryotic cell lines
☒	☐	Palaeontology and archaeology
☐	☒	Animals and other organisms
☒	☐	Human research participants
☒	☐	Clinical data
☒	☐	Dual use research of concern

Methods

n/a	Involved in the study	
☒	☐	ChIP-seq
☐	☒	Flow cytometry
☒	☐	MRI-based neuroimaging

Antibodies

Antibodies used

The antibody list includes the target, fluorochrome, clone name, company, cat.no., dilution, and the link to the datasheet:
CD45, PE, HI30, Biolegend, 304008, 1:20, https://www.biolegend.com/en-us/products/pe-anti-human-cd45-antibody-708?GroupID=BLG5926
IgG1, PE, MOPC-21, Biolegend, 400111, 1:20, https://www.biolegend.com/en-us/products/pe-mouse-igg1-kappa-isotype-ctrl-1408
CD3, AF488, UCHT1, Biolegend, 300415, 1:20, https://www.biolegend.com/en-us/products/alexa-fluor-488-anti-human-cd3-antibody-2726
IgG1, AF488, MOPC-21, Biolegend, 400129, 1:20, https://www.biolegend.com/en-us/products/alexa-fluor-488-mouse-igg1-kappa-isotype-ctrl-fc-2687
CD19, PerCP, HIB19, Biolegend, 302228, 1:20, https://www.biolegend.com/en-us/products/percp-anti-human-cd19-antibody-4225
IgG1, PerCP, MOPC-21, Biolegend, 400148, 1:20, https://www.biolegend.com/en-us/products/percp-mouse-igg1-kappa-isotype-ctrl-4204
CD33, BV605, P67.6, Biolegend, 366612, 1:20, https://www.biolegend.com/en-us/products/brilliant-violet-605-anti-human-cd33-antibody-12255
IgG1, BV605, MOPC-21, Biolegend, 400162, 1:20, https://www.biolegend.com/en-us/products/brilliant-violet-605-mouse-igg1-kappa-isotype-ctrl-7630
CD7, APC, 4H9/CD7, Biolegend, 395605, 1:20, https://www.biolegend.com/en-us/products/apc-anti-human-cd7-antibody-20182?GroupID=GROUP28
IgG2a, APC, MOPC-173, Biolegend, 400219, 1:20, https://www.biolegend.com/en-us/products/apc-mouse-igg2a-kappa-isotype-

ctrl-1397

CD56, PerCP/Cy5.5, MEM-188, Biolegend, 304625, 1:20, https://www.biolegend.com/en-us/products/percp-cyanine5-5-anti-human-cd56-ncam-antibody-6809
IgG2a, PerCP/Cy5.5, MOPC-173, Biolegend, 400251, 1:20, https://www.biolegend.com/en-us/products/percp-cyanine5-5-mouse-igg2a-kappa-isotype-ctrl-4207
F4/80, APC, QA17A29, Biolegend, 157305, 1:40, https://www.biolegend.com/en-us/products/apc-anti-mouse-f4-80-recombinant-antibody-18756?GroupID=GROUP20
IgG1, APC, MOPC-21, Biolegend, 400119, 1:20, https://www.biolegend.com/en-us/products/apc-mouse-igg1-kappa-isotype-ctrl-1404
CD68, FITC, Y1/82A, Biolegend, 333805, 1:20, https://www.biolegend.com/en-us/products/fitc-anti-human-cd68-antibody-4844
IgG2b, FITC, MPC-11, Biolegend, 400309, 1:20, https://www.biolegend.com/en-us/products/fitc-mouse-igg2b-kappa-isotype-ctrl-1412
anti mouse CD45 , AF700, I3/2.3, Biolegend, 147715, 1:50, https://www.biolegend.com/en-us/products/alexa-fluor-700-anti-mouse-cd45-antibody-16471
IgG2b, AF700, RTK4530, Biolegend, 400628, 1:20, https://www.biolegend.com/en-us/products/alexa-fluor-700-rat-igg2b-kappa-isotype-ctrl-3381

HLA-E , APC, 3D12, Biolegend, 342605, 1:20, https://www.biolegend.com/en-us/products/apc-anti-human-hla-e-antibody-10760
IgG1, APC, MOPC-21, Biolegend, 400121, 1:20, https://www.biolegend.com/en-us/products/apc-mouse-igg1-kappa-isotype-ctrl-fc-3034
HLA-G , APC, 87G, Biolegend, 335909, 1:20, https://www.biolegend.com/en-us/products/apc-anti-human-hla-g-antibody-7117
IgG2a, APC, MOPC-173, Biolegend, 400221, 1:20, https://www.biolegend.com/en-us/products/apc-mouse-igg2a-kappa-isotype-ctrl-fc-3044
PD-L1 , PE, 29E.2A3, Biolegend, 329705, 1:20, https://www.biolegend.com/en-us/products/pe-anti-human-cd274-b7-h1-pd-l1-antibody-4375
IgG2b, PE, MPC-11, Biolegend, 400313, 1:20, https://www.biolegend.com/en-us/products/pe-mouse-igg2b-kappa-isotype-ctrl-1414

SIRPa, APC, 15-414, Biolegend, 372105, 1:20, https://www.biolegend.com/en-us/products/apc-anti-human-cd172a-sirpalpha-antibody-14165
IgG2a, APC, MOPC-173, Biolegend, 400219, 1:20, https://www.biolegend.com/en-us/products/apc-mouse-igg2a-kappa-isotype-ctrl-1397
CD94, FITC, DX22, Biolegend, 305504, 1:20, https://www.biolegend.com/en-us/products/fitc-anti-human-cd94-antibody-647
IgG1, FITC, MOPC-21, Biolegend, 400107, 1:20, https://www.biolegend.com/en-us/products/fitc-mouse-igg1-kappa-isotype-ctrl-1406
ILT2, APC, GHI/75, Biolegend, 333719, 1:20, https://www.biolegend.com/en-us/products/apc-anti-human-cd85j-ilt2-antibody-15530
IgG2b, APC, MPC-11, Biolegend, 400321, 1:20, https://www.biolegend.com/en-us/products/apc-mouse-igg2b-kappa-isotype-ctrl-1410
PD-1 , FITC, EH12.2H7, Biolegend, 329903, 1:20, https://www.biolegend.com/en-us/products/fitc-anti-human-cd279-pd-1-antibody-4411
IgG1, FITC, MOPC-21, Biolegend, 400107, 1:20, https://www.biolegend.com/en-us/products/fitc-mouse-igg1-kappa-isotype-ctrl-1406

HLA-A,B,C , APC, G46_2.6, BD Biosciences, 555555, 1:20, https://www.bdbiosciences.com/en-us/products/reagents/flow-cytometry-reagents/research-reagents/single-color-antibodies-ruo/apc-mouse-anti-human-hla-abc.555555
IgG1 , APC, MOPC-21, BD Biosciences, 554681, 1:5, https://www.bdbiosciences.com/en-us/products/reagents/flow-cytometry-reagents/research-reagents/flow-cytometry-controls-and-lysates/apc-mouse-igg1-isotype-control.554681
HLA-DR,DP,DQ , AF647, Tu39, BD Biosciences, 563591, 1:20, https://www.bdbiosciences.com/en-us/products/reagents/flow-cytometry-reagents/research-reagents/single-color-antibodies-ruo/alexa-fluor-647-mouse-anti-human-hla-dr-dp-dq.563591
IgG2a, AF647, G155-178, BD Biosciences, 565357, 1:60, https://www.bdbiosciences.com/en-us/products/reagents/flow-cytometry-reagents/research-reagents/flow-cytometry-controls-and-lysates/alexa-fluor-647-mouse-igg2a-isotype-control.565357
CD47 , FITC, CC2C6, Biolegend, 323106, 1:20, https://www.biolegend.com/en-us/products/fitc-anti-human-cd47-antibody-3707
IgG1, FITC, MOPC-21, Biolegend, 400110, 1:20, https://www.biolegend.com/en-us/products/fitc-mouse-igg1-kappa-isotype-ctrl-fc-3036
CD47 , PerCP/Cy5.5, B6H12, BD Biosciences, 561261, 1:20, https://www.bdbiosciences.com/en-us/products/reagents/flow-cytometry-reagents/research-reagents/single-color-antibodies-ruo/percp-cy-5-5-mouse-anti-human-cd47.561261
IgG1, PerCP/Cy5.5, MOPC-21, BD Biosciences, 550795, 1:20, https://www.bdbiosciences.com/en-us/products/reagents/flow-cytometry-reagents/research-reagents/flow-cytometry-controls-and-lysates/percp-cy-5-5-mouse-igg1-isotype-control.550795
CD47 , PE, CC2C6, Biolegend, 323108, 1:20, https://www.biolegend.com/en-us/products/pe-anti-human-cd47-antibody-3708

C-Peptide, n/a, polyclonal, Novus Biologicals, MAB14171, 1:20, https://www.novusbio.com/products/c-peptide-antibody-790904_mab14171
NKX6.1, n/a, polyclonal, Novus Biologicals, NBP1-49672, 1:20, https://www.novusbio.com/products/nkx61-antibody_nbp1-49672
CHGA, n/a, polyclonal, Novus Biologicals, NB120-15160, 1:20, https://www.novusbio.com/products/chromogranin-a-antibody_nb120-15160
anti-rat Ig, AF488, polyclonal, Thermo Fisher, A-11006, 1:500, https://www.thermofisher.com/antibody/product/Goat-anti-Rat-IgG-H-L-Cross-Adsorbed-Secondary-Antibody-Polyclonal/A-11006?gclid=Cj0KCQjwtsCgBhDEARIsAE7RYh0Febhf3AJHIzWTLZiwqQauCac7Dik8YRc8sEh5Uly92b61H6ofjGIaAuAEEALw_wcB&ef_id=Cj0KCQjwtsCgBhDEARIsAE7RYh0Febhf3AJHIzWTLZiwqQauCac7Dik8YRc8sEh5Uly92b61H6ofjGIaAuAEEALw_wcB:G:s&s_kwcid=AL!3652!3!444085820557!e!!g!!anti%20rat%20488!596889499!104823981722&cid=bid_pca_aus_r01_co_cp1359_pjt0000_bid00000_0se_gaw_nt_pur_con
anti-mouse Ig, AF647, polyclonal, Thermo Fisher, A-21235, 1:500, https://www.thermofisher.com/antibody/product/Goat-anti-Mouse-IgG-H-L-Cross-Adsorbed-Secondary-Antibody-Polyclonal/A-21235
anti-rabbit Ig, AF647, polyclonal, Thermo Fisher, A-21244, 1:500, https://www.thermofisher.com/antibody/product/Goat-anti-Rabbit-IgG-H-L-Cross-Adsorbed-Secondary-Antibody-Polyclonal/A-21244

Islet 1, n/a, EPR10362, abcam, ab178400, 1:250, https://www.abcam.com/products/primary-antibodies/islet-1-antibody-epr10362-ab178400.html

Insulin , AF647, 2D11-H5, Santa Cruz Biotechnology, sc-8033 AF647, 1:20, https://www.scbt.com/p/insulin-antibody-2d11-h5
IgG1 κ , AF647, MOPC-21, Biolegend, 400130, 1:20, https://www.biolegend.com/en-us/products/alexa-fluor-647-mouse-igg1-kappa-isotype-ctrl-fc-2688
Glucagon, PE, C-11, Santa Cruz Biotechnology, sc-514592 PE, 1:20, https://www.scbt.com/p/glucagon-antibody-c-11?requestFrom=search
IgG1 κ, PE, MOPC-21, Biolegend, 400113, 1:20, https://www.biolegend.com/en-us/products/pe-mouse-igg1-kappa-isotype-ctrl-fc-3035
Somatostatin, FITC, G-10, Santa Cruz Biotechnology, sc-55565 FITC, 1:20, https://www.scbt.com/p/somatostatin-antibody-g-10?requestFrom=search
IgG2b κ, FITC, MPC-11, Biolegend, 400309, 1:20, https://www.biolegend.com/en-us/products/fitc-mouse-igg2b-kappa-isotype-ctrl-1412

CD8, FITC, LT8, abcam, ab28010, 1:5, https://www.abcam.com/products/primary-antibodies/fitc-cd8-antibody-lt8-ab28010.html
NKG2A, PE, REA110, Miltenyi, 130-114-092, 1:50, https://www.miltenyibiotec.com/US-en/products/cd159a-nkg2a-antibody-anti-human-reafinity-rea110.html#gref

CD68, unconjugated, KP1, Abcam, ab955, 1:60,000, https://www.abcam.com/products/primary-antibodies/cd68-antibody-kp1-ab955.html
CD3, unconjugated, SP7, Abcam, ab16669, 1:400, https://www.abcam.com/products/primary-antibodies/cd3-antibody-sp7-ab16669.html
CD20, unconjugated, polyclonal, Thermo Fisher, PA5-16701, 1:800, https://www.thermofisher.com/antibody/product/CD20-Antibody-Polyclonal/PA5-16701

Validation

Each antibody was tested with positive and negative control prior to staining the samples. Antibody concentration were gathered from vendors datasheet. Isotype and tested antibody were concentration matched.

Eukaryotic cell lines

Policy information about cell lines

Cell line source(s)

The Human Episomal iPSC Line was purchased from Thermo Fisher Scientific (Waltham, MA). Human K562 were purchased from ATCC. Human NK-cells were purchased from StemCell Technologies. Rhesus macaque iPSCs were generated and provided by Dr. Cynthia E. Dunbar, M.D., NHLBI. The generation of rhesus macaque iPSCs was described in Hong SG. Cell Reports. 2014;7:1298-1309.

Authentication

None of the cell lines used have been authenticated.

Mycoplasma contamination

All cell lines were tested and negative for mycoplasma contamination using the Universal Mycoplasma test kit from ATCC.

Commonly misidentified lines
(See ICLAC register)

No commonly misidentified cell lines were used.

Animals and other organisms

Policy information about studies involving animals; ARRIVE guidelines recommended for reporting animal research

Laboratory animals

Male NSG mice (strain 005557) and female humanized NSG-SGM3 mice (strain 013062) were purchased from the Jackson Laboratories and used as recipients for different assays. All rhesus macaque monkeys were purchased from Alpha Genesis Inc. Six female and 16 male rhesus macaques (3-4 kg) were used.

Wild animals

No wild animals were used

Field-collected samples

No field collected samples were used

Ethics oversight

Mice received humane care in compliance with the IACUC and performed according to California's guidelines. Monkey procedures were approved by the Alpha Genesis Inc. IACUC and regulated by USDA.

Note that full information on the approval of the study protocol must also be provided in the manuscript.

Flow Cytometry

Plots

Confirm that:

☒ The axis labels state the marker and fluorochrome used (e.g. CD4-FITC).

☒ The axis scales are clearly visible. Include numbers along axes only for bottom left plot of group (a 'group' is an analysis of identical markers).

☒ All plots are contour plots with outliers or pseudocolor plots.

☒ A numerical value for number of cells or percentage (with statistics) is provided.

Methodology

Sample preparation	iPSCs and K562 were counted, stained and measured as single cell suspension in PBS+2% FCS hi. PBMCs were enriched from rhesus whole blood by Ficoll separation. Cells were counted and stained in PBS+2% FCS hi. Splenocytes of humanized mice were isolated from the spleen. Cells were counted and stained in PBS+2% FCS hi.
Instrument	The BD Aria Fusion (BD Bioscience) or Attune (Thermo Fisher) were used.
Software	The FACSDiva 8.0.3 software or Attune NxT software were used.
Cell population abundance	For flow cytometry analysis, more than 10,000 positive cells were measured. Cell sorting was gated for the desired population and sorted for the cell amount needed for assays.
Gating strategy	Samples were gated in FSC/SSC for the correct cell size and live cells. Isotype was measured for each sample as defined as unspecific staining threshold.

☒ Tick this box to confirm that a figure exemplifying the gating strategy is provided in the Supplementary Information.
